# Effects of preoperative carbohydrate loading on recovery after elective surgery: A systematic review and Bayesian network meta-analysis of randomized controlled trials

**DOI:** 10.3389/fnut.2022.951676

**Published:** 2022-11-23

**Authors:** Enyu Tong, Yiming Chen, Yanli Ren, Yuanyuan Zhou, Chunhong Di, Ying Zhou, Shihan Shao, Shuting Qiu, Yu Hong, Lei Yang, Xiaohua Tan

**Affiliations:** ^1^School of Public Health, Hangzhou Normal University, Hangzhou, China; ^2^The Affiliated Hospital of Hangzhou Normal University, Hangzhou Normal University, Hangzhou, China

**Keywords:** preoperative carbohydrate loading, insulin resistance, postoperative comfort and safety, elective surgery, adults, Bayesian network meta-analysis

## Abstract

**Background:**

Preoperative carbohydrate loading is an important element of the enhanced recovery after surgery (ERAS) paradigm in adult patients undergoing elective surgery. However, preoperative carbohydrate loading remains controversial in terms of improvement in postoperative outcomes and safety. We conducted a Bayesian network meta-analysis to evaluate the effects and safety of different doses of preoperative carbohydrates administrated in adult patients after elective surgery.

**Methods:**

MEDLINE (PubMed), Web of Science, EMBASE, EBSCO, the Cochrane Central Register of Controlled Trials, and China National Knowledge Infrastructure (CNKI) were searched to identify eligible trials until 16 September 2022. Outcomes included postoperative insulin resistance, residual gastric volume (RGV) during the surgery, insulin sensitivity, fasting plasma glucose (FPG), fasting serum insulin (Fin) level, the serum levels of C-reactive protein (CRP), postoperative scores of pain, patients’ satisfaction, thirst, hunger, anxiety, nausea and vomit, fatigue, and weakness within the first 24 h after surgery and the occurrences of postoperative infection. The effect sizes were estimated using posterior mean difference (continuous variables) or odds ratios (dichotomous variables) and 95 credible intervals (CrIs) with the change from baseline in a Bayesian network meta-analysis with random effect.

**Results:**

Fifty-eight articles (*N* = 4936 patients) fulfilled the eligibility criteria and were included in the meta-analysis. Both preoperative oral low-dose carbohydrate loading (MD: –3.25, 95% CrI: –5.27 to –1.24) and oral high-dose carbohydrate loading (MD: –2.57, 95% CrI: –4.33 to –0.78) were associated with postoperative insulin resistance compared to placebo/water. When trials at high risk of bias were excluded, association with insulin resistance was found for oral low-dose carbohydrate loading compared with placebo/water (MD: –1.29, 95%CrI: –2.26 to –0.27) and overnight fasting (MD: –1.17, 95%CrI: –1.88 to –0.43). So, there was large uncertainty for all estimates vs. control groups. In terms of safety, oral low-dose carbohydrate administration was associated with the occurrences of postoperative infection compared with fasting by 0.42 (95%Crl: 0.20–0.81). In the other outcomes, there was no significant difference between the carbohydrate and control groups.

**Conclusion:**

Although preoperative carbohydrate loading was associated with postoperative insulin resistance and the occurrences of postoperative infection, there is no evidence that preoperative carbohydrate administration alleviates patients’ discomfort.

**Systematic review registration:**

[https://www.crd.york.ac.uk/PROSPERO/], identifier [CRD42022312944].

## Introduction

Surgery, as a form of stress, induces peripheral insulin resistance, which can result in hyperglycemia, which, in turn, may have potentially adverse effects on postoperative patients (1, 2). Efficient management of preoperative interventions could reduce postoperative complications and facilitate recovery.

Enhanced recovery after surgery (ERAS) is a multimodal, multidisciplinary project aimed at improving the recovery of patients undergoing surgery during the entire perioperative period (3). The overall complication occurrences were reduced by up to 50% when the ERAS protocols were used compared with traditional perioperative patient management (4, 5).

The preoperative administration of carbohydrate loading as a part of ERAS protocols reduces insulin resistance and tissue glycosylation, improves postoperative glucose control, and enhances postoperative comfort (6). Several randomized controlled trials (RCTs) and meta-analysis have shown that preoperative carbohydrate loading decreased postoperative insulin resistance and side effects compared with those consuming placebo/water or in a fasted state (7, 8). Other RCTs, however, have shown that perioperative carbohydrate administration had no effect on postoperative insulin resistance (9, 10). Thus, the administration of preoperative carbohydrates remains somewhat controversial.

The conventional pairwise meta-analysis has its limitations. First, the previous meta-analysis cannot compare different controls (such as fasting, placebo, or water) simultaneously, so these meta-analyses need to combine these groups into one treatment arm, thus limited interpretability (8). Second, because of the scarcity of direct head-to-head comparisons of interventions in trials, it is unable to assess the comparative effects of interventions (11).

Therefore, to overcome this limitation, we conducted an updated systematic review and network meta-analysis (NMA) to pool and analyze data comparing different preoperative drinks used for clinical and metabolic postoperative outcomes in adult patients undergoing elective surgery (12).

## Materials and methods

### Protocol registration

This is a systematic review and NMA of preoperative carbohydrate intervention trials in adult patients undergoing elective surgery. The Preferred Reporting Items for Systematic Reviews (PRISMA) and Meta-analyses for RCTs were used to organize the reporting (13). The study protocol was registered (registration number: CRD42022312944) with the International Prospective Register of Systematic Reviews (PROSPERO) following the standard reporting method.

### Data sources

MEDLINE (PubMed), Web of Science, EMBASE, EBSCO, the Cochrane Central Register of Controlled Trials, and China National Knowledge Infrastructure (CNKI) were searched to identify eligible trials. We updated the literature search weekly, and the search was performed from database inception until 16 September 2022 (details are shown in Supplementary Table 1).

### Trial selection criteria

Eligible trials included the preoperative administration of at least 10 g carbohydrate loading (orally or intravenously) before 4 h of the surgery started, and with fasting, placebo, or water, undergoing any type of elective surgery in adults. Studies also included carbohydrate-based solutions containing other compounds (such as glutamine and whey protein). Patients with diabetes mellitus or those who were receiving emergency surgery were also excluded.

### Trial identification

Two investigators independently screened articles by title, abstract, and full text using the inclusion criteria. The inclusion of a study was decided by consensus between the two investigators. When differences occurred, investigators consulted or discussed with a third one to solve them.

### Intervention categories

Five categories were used to classify the preoperative administration for the included RCTs:

(1) Low-dose carbohydrate: The dose of oral carbohydrate is between 10 and 50 g before surgery (10–50 g);
(2) High-dose carbohydrate: The dose of oral carbohydrate is greater than 50 g before surgery (>50 g);
(3) Carbohydrate, iv: preoperative carbohydrate by intravenous perfusion;
(4) Placebo/water (control group);
(5) Fasting (control group).

### Outcome measures

The primary outcome was mean change from baseline to the end point (within the first 24 h after surgery) in insulin resistance, as measured by the Homeostasis Model Assessment of Insulin Resistance (HOMA-IR) method according to the following equation: HOMA-IR = [fasting insulin(μU/mL) × fasting glucose (mmol/L)]/22.5)]. Secondary outcomes were included: residual gastric volume (RGV) during the operation; insulin sensitivity (measured by the hyperinsulinemic glucose clamp method) within the first 24 h after surgery; fasting plasma glucose (FPG) within the first 24 h after surgery; fasting serum insulin (Fin) level within the first 24 h after surgery; the serum levels of C-reactive protein (CRP) within the first 24 h after surgery; postoperative pain, patients’ satisfaction, thirst, hunger, anxiety, postoperative nausea and vomit (PONV), fatigue, weakness (all measured on a visual analog scale [VAS]), and the occurrences of postoperative infection.

### Data extraction

The following study characteristics were extracted for each eligible study: (1) trial information: the first author, study year, the study country, and trial name; (2) patient characteristics: sample size in each treatment, the type of surgery, and American Society of Anesthesiologists (ASA) grade; (3) intervention details: the type, total dose, administrate route, and timing of each treatment; (4) outcome measures: the primary or secondary outcomes including insulin resistance, RGV, insulin sensitivity, FPG, Fin level, the serum levels of CRP, pain, thirst, hunger, anxiety, nausea and vomit, fatigue, weakness within the first 24 h after surgery, and the occurrences of postoperative infection.

### Quality and risk of bias assessment

The quality of every eligible trial was assessed independently by two researchers based on the Cochrane risk of bias 2.0 tool in RCTs in a blind fashion (14), which contains five domains: randomization process, deviations from the intended interventions, missing outcome data, measurement of the outcome, and selection of the reported result. Disagreements were discussed and resolved through consensus.

### Data synthesis and analysis

We estimated the effect sizes for group differences with respect to baseline changes. We used the imputation of correlation when standard deviations were not available for the mean change value, but were available for baseline and endpoint values (15). Arithmetic difference between baseline and end point was used when the study did not report mean change. Meta-analytic calculations were conducted using R Version 4.1.2 (RStudio, Boston, MA, USA) (16). We performed a Bayesian network model and all analyses were conducted using the “gemtc” package version 1.0-1 (17) and jagsUI packages version 1.5.2 (18). Network plot command of Stata version 16.0 (StataCorp, College Station, TX 77845, USA) was used to draw the comparison-adjusted funnel (19).

Mean difference (MD) was used to model continuous variables, whereas dichotomous outcomes were modeled using a binomial likelihood and logit link (20). The outcomes were converted to standard units. Additionally, missing standard deviations were calculated from standard errors, ranges, or interquartile ranges as described in the Cochrane Handbook (21). In this study, a NMA was conducted within a Bayesian framework to assess the relative effectiveness of preoperative carbohydrate loading for recovery after elective surgery.

The consistency model and the inconsistency model were used to analyze all outcomes, and the difference in deviance information criterion (DIC) and *I*^2^ was used to compare the overall findings. If the difference in DIC between the two models was ≥ 5, we used the inconsistency model. Both a fixed-effect model and a random model were run for each result, and a lower DIC value indicated a greater model fit.

The Markov chain Monte Carlo (MCMC) algorithm was used to estimate the posterior densities of all unknown parameters in each model. It was based on simulations of 200,000 iterations in each of four chains and provided evidence for confirming the convergence of the models.

The trials we included were tested for consistency and inconsistency. We used the node splitting method to perform to compare the treatment effect direct and indirect comparisons of multiple interventions, and *P* > 0.05 was considered to indicate good consistency (22, 23).

Probability values were summarized and are reported as the surface under the cumulative ranking (SUCRA) curve. When the intervention was certain to be the worst, the SUCRA value would be 0, and when it was certain to be the best, the SUCRA value would be 1 (24).

To investigate the source of heterogeneity, meta-regression was used to explore and account for the heterogeneity with the risk of bias, the category of surgery, and the blinding of these studies’ designs.

The planned sensitivity analyses of the outcomes were conducted to evaluate the robustness of the model. First, in addition to the Bayesian random effect network, sensitivity analyses were performed using a fixed-effect network. Second, the transitivity assumption was tested by splitting the “water or placebo” group within the network. Third, all analyses were repeated after excluding high-risk trials and data from imputation methods. In addition, for the primary outcome, we planned to add subgroup analyses conducted for different surgical categories, and a comparison-adjusted funnel plot was used to assess the presence of small-study effects bias.

The Confidence in Network Meta-Analysis (CINeMA) methodological framework and application were used to evaluate confidence in NMA effect estimates for all outcomes and treatment comparisons (25, 26).

## Results

### Study selection

**FIGURE 1:**
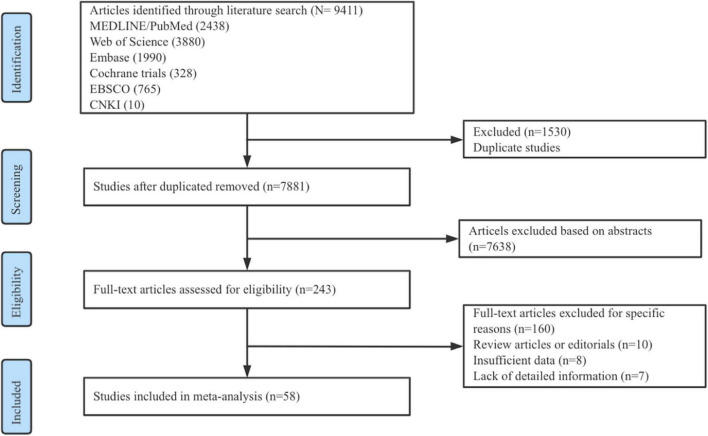
Flow diagram of study identification, screening, eligibility assessment, and inclusion.

A total of 9411 records were retrieved, of which 58 articles (*N* = 4936 patients) fulfilled the eligibility criteria and were included in the meta-analysis, the retrieval process is shown in Figure 1. A total of five interventions were included in this meta-analysis: oral low-dose carbohydrate (10–50 g) loading, oral high-dose carbohydrate (more than 50 g) loading, carbohydrate by intravenous perfusion (Carbohydrate, iv), placebo/water, and fasting. Detailed trial and patient characteristics are shown in Table 1.

**TABLE 1:** Characteristics of the included studies.

							Type of intervention			
References	Country	Sample size (I/C)	Type of surgery	ASA	Type of study	Type	Specification, %, and route	Dose, ml	Comparator	Outcomes
Ajuzieogu et al. (52)	Nigeria	30/30/30	Abdominal myomectomy	I–II	RCT	High-dose carbohydrate	150 g, oral	‡1200	Placebo; fasting	➀ ➂
Bisgaard et al. (67)	Denmark	43/43	Laparoscopic cholecystectomy	I–II	RCT	High-dose carbohydrate	150 g, oral	‡1200	Water	➁ ⑬
Braga et al. (53)	Italy	18/18	Pancreaticoduodenectomy	N.S	RCT	Low-dose carbohydrate	50 g, oral	*250	Placebo	➀ ➇
Breuer et al. (74)	Germany	56/60/44	Cardiac surgery	III–IV	RCT	High-dose carbohydrate	150 g, oral	‡1200	Placebo; fasting	⑮
Canbay et al. (27)	Turkey	25/25	Pancreaticoduodenectomy	I–II	RCT	High-dose carbohydrate	150 g, oral	‡1200	Fasting	➆
Chaudhary et al. (70)	Nepal	33/33	Femur fracture surgery	N.S	RCT	High-dose carbohydrate	150 g, oral	‡1200	Fasting	➁
Chen et al. (28)	China	12/12/12	Open gastrectomy for cancer	I–II	RCT	Low-dose carbohydrate	50 g, oral	*500	Water; fasting	➀ ➆
Cho et al. (29)	Korea	44/44	Laparoscopic Gynecologic Surgery	I–II	RCT	High-dose carbohydrate	76.8 g, oral	**600	Fasting	➁ ➄ ➅ ➆ ⑮
Borges Dock-Nascimento et al. (56)	Brazil	12/12/12	Laparoscopic cholecystectomy	I–II	RCT	High-dose carbohydrate	75 g, oral	**600	Water; fasting	➄
Doo et al. (57)	Korea	25/25	Thyroidectomy	I–II	RCT	High-dose carbohydrate	51.2 g, oral	*400	Fasting	➂ ➄ ➈ ➉ ⑪ ⑫ ⑬
Faria et al. (30)	Brazil	11/10	Laparoscopic cholecystectomy	I–II	RCT	Low-dose carbohydrate	25 g, oral	*200	Fasting	➄ ➅ ➆
Feguri et al. (31)	Brazil	20/20	CABG	N.S	RCT	High-dose carbohydrate	75 g, oral	**600	Water	➆
Feguri et al. (75)	Brazil	14/14	CABG	N.S	RCT	Low-dose carbohydrate	25 g, oral	*200	Fasting	⑮
Gianotti et al. (54)	Italy	331/331	Major abdominal surgery	I–III	RCT	High-dose carbohydrate	100 g, oral	†††800	Water	➀ ⑮
Gümüs et al. (32)	Turkey	35/33	Laparoscopic cholecystectomy	N.S	RCT	Low-dose carbohydrate	50 g, oral	*400	Fasting	➄ ➆
Harsten et al. (72)	Sweden	30/30	Hip replacement	I–III	RCT	High-dose carbohydrate	100 g, oral	¶ 800	Placebo	⑫
He et al. (48)	China	30/29/29	Elective cesarean delivery	N.S	RCT	Low-dose carbohydrate	50 g, oral	*400	Placebo; fasting	➄ ➅ ➆
Helminen et al. (68)	Finland	57/56	Laparoscopic cholecystectomy	I–II	RCT	High-dose carbohydrate	67 g, oral	*200	Fasting	➁ ➉ ⑪ ⑫ ⑬
Hosny et al. (65)	UK	21/21	CABG	II–III	RCT	Low-dose carbohydrate	50 g, iv	500	Water	➄ ➅
Itou et al. (55)	Japan	135/139	Mixed#	I–II	RCT	Low-dose carbohydrate	25 g, oral	¶¶¶ 1000	Fasting	➀
Järvelä et al. (79)	Finland	50/51	CABG	N.S	RCT	Low-dose carbohydrate	50 g, oral	*400	Fasting	➅
Kaska et al. (58)	Czech Republic	75/72/74	Colorectal surgery	I–II	RCT	High-dose carbohydrate; Carbohydrate, iv	100.8 g, oral; 50 g, iv	¶ 800; *500	Fasting	➄ ⑮
Kweon et al. (33)	Korea	43/45	Orthopedic surgery	I–III	RCT	High-dose carbohydrate	102 g, oral	¶ 800	Fasting	➄ ➅ ➆
Lauwick et al. (69)	Belgium	100/100	Thyroidectomy	I–II	RCT	Low-dose carbohydrate	50 g, oral	*400	Placebo	➁ ➈ ➉ ⑪ ⑬
Lee et al. (80)	Republic of Korea	28/29	CABG	N.S	RCT	High-dose carbohydrate	102 g, oral	¶ 800	Fasting	➄
Ljungqvist et al. (81)	Sweden	6/6	Open cholecystectomy	I–III	RCT	High-dose carbohydrate, iv	250 g, iv	N.S	Fasting	➃
Ljunggren and Hahn (63)	Sweden	19/18/20	Hip replacement surgery	I–III	RCT	High-dose carbohydrate	150 g, oral	‡1200	Water; fasting	➃ ➄
Ljunggren et al. (64)	Sweden	10/12	Hip replacement surgery	I–III	RCT	High-dose carbohydrate	150 g, oral	‡1200	Flavored water	➃
Liu et al. (59)	China	58/62	Craniotomy	I–II	RCT	Low-dose carbohydrate	50 g, oral	*400	Fasting	➄ ⑮
Liu et al. (73)	China	60/60	Open gastrointestinal surgery	II–IV	RCT	Low-dose carbohydrate	25 g, oral	*200	Fasting	⑮
Mathur et al. (9)	New Zealand	69/73	Abdominal surgery	I–III	RCT	High-dose carbohydrate	150 g, oral	‡1200	Placebo	➄ ➅ ➇
Marquini et al. (34)	Brazil	34/40	Gynecologic surgery	I–II	RCT	High-dose carbohydrate	178 g, oral	¶¶ 200	Placebo	➄ ➅ ➆
Mousavie et al. (62)	Iran	26/26/26	Laparoscopic cholecystectomy	I–II	RCT	Low-dose carbohydrate; Carbohydrate, iv	25 g, oral; 25 g, i.v	*200; *250	Fasting	➁ ➄ ⑫
Nygren et al. (60)	Sweden	7/7	Colorectal surgery	N.S	RCT	High-dose carbohydrate	150 g, oral	‡1200	Fasting	➄
Onalan et al. (35)	Turkey	25/25	Laparoscopic cholecystectomy	N.S	RCT	High-dose carbohydrate	150 g, oral	‡1200	Fasting	➁ ➄ ➆ ➈ ➉ ⑪
Pexe-Machado et al. (38)	Brazil	10/12	Laparotomy for gastrointestinal malignancy##	I–III	RCT	High-dose carbohydrate	66 g, oral	**600	Fasting	➄ ➅ ➆ ➇
Pêdziwiatr et al. (36)	Cracow	20/20	Laparoscopic cholecystectomy	I–III	RCT	High-dose carbohydrate	50.4 g, oral	*400	Water	➄ ➅ ➆ ⑮
Perrone et al. (37)	Brazil	8/9	Cholecystectomy^ or inguinal hernia repair	I–II	RCT	High-dose carbohydrate	54 g, oral	††711	Water	➄ ➅ ➆ ➇
Rapp-Kesek et al. (39)	Sweden	9/9	CABG	N.S	RCT	High-dose carbohydrate	100 g, oral	†800	Fasting	➄ ➅ ➆
Qin et al. (49)	China	111/112	Elective gastrectomy, colorectal resection, or duodenopancreatectomy	N.S	RCT	High-dose carbohydrate	150 g, oral	‡1200	Water	➄ ➅ ➆ ⑮
de Andrade Gagheggi Ravanini et al. (40)	Brazil	21/17	Cholecystectomy	I–II	RCT	High-dose carbohydrate	67 g, oral	*200	Fasting	➅ ➆ ⑫
Rizvanović et al. (41)	Croatia	25/25	Colorectal surgery	I–III	RCT	High-dose carbohydrate	75 g, oral	**600	Fasting	➄ ➅ ➆ ➇ ➈
Sada et al. (71)	Kosovo	22/23/26	Abdominal surgery	I–II	RCT	High-dose carbohydrate	150 g, oral	‡1200	Placebo; fasting	➈ ➉ ⑪ ⑫ ⑭
Awad et al. (82)	UK	20/20	Laparoscopic cholecystectomy	N.S	RCT	Low-dose carbohydrate	45 g, oral	***900	Placebo	➄
Singh et al. (46)	India	40/40/40	Laparoscopic cholecystectomy	N.S	RCT	High-dose carbohydrate	75 g, oral	**600	Placebo; fasting	➄ ➅ ➆
Shi et al. (43)	China	25/25/25	Cesarean section	I–II	RCT	Low-dose carbohydrate	42.6 g, oral	*300	Water; fasting	➄ ➅ ➆
Soop et al. (7)	Sweden	8/7	Hip replacement surgery	N.S	RCT	High-dose carbohydrate	150 g, oral	‡1200	Placebo	➃ ➄ ➅
Soop et al. (83)	Sweden	8/6	Hip replacement surgery	I–II	RCT	High-dose carbohydrate	150 g, oral	‡1200	Placebo	➃ ➄
van Stijn et al. (84)	Netherlands	10/8	Rectal cancer surgery	N.S	RCT	Low-dose carbohydrate	42 g, oral	‡‡‡750	Placebo	➃ ➄ ➇
Suh et al. (85)	USA	70/64	Mixed^^	II–IV	RCT	High-dose carbohydrate	100 g, oral	†††592	Fasting	➄
Tewari et al. (86)	UK	16/16	Elective major open abdominal surgery	N.S	RCT	High-dose carbohydrate	150 g, oral	‡1200	Placebo	➃
Tran et al. (47)	Canada	19/19	Mixed###	N.S	RCT	Low-dose carbohydrate	50 g, oral	§§ 400	Fasting	➆ ⑮
Wang et al. (87)	China	36/37	Endoscopic submucosal dissection	I–II	RCT	Carbohydrate	42.6 g, oral	§§§ 1065	Fasting	➈ ➉ ⑫ ⑬ ⑮
Wu et al. (50)	China	43/43	Free flap surgery for oral cancer	I–III	RCT	Low-dose carbohydrate	48 g, oral	*400	Fasting	➄ ➅ ➆ ⑮
Yi et al. (66)	Malaysia	62/56	Mixed^^^	I–III	RCT	Low-dose carbohydrate	27 g, oral	††711	Fasting	➇ ⑮
Yu et al. (42)	China	24/24	Radical distal subtotal gastrectomy	I–III	RCT	Low-dose carbohydrate	50 g, oral	§§ 500	Placebo	➄ ➅ ➆
Yuill et al. (61)	UK	31/34	Abdominal surgery	N.S	RCT	High-dose carbohydrate	151.2 g, oral	‡1200	§ Placebo	➄ ➅
Zhang and Min (44)	China	29/29	Gynecological surgery	I–II	RCT	High-dose carbohydrate	150 g, oral	‡1200	Fasting	➁ ➄ ➅ ➆ ➇ ➈ ➉ ⑭
Zhou (45)	China	29/30	Gastrectomy	N.S	RCT	Low-dose carbohydrate	50 g, oral	*500	Fasting	➄ ➅ ➆ ⑮

Outcomes: ➀: residual gastric volume (RGV) during the surgery; ➁: postoperative pain; ➂: postoperative patient satisfaction; ➃: insulin sensitivity (measured by hyperinsulinemic glucose clamp); ➄: postoperative fasting plasma glucose (FPG); ➅: postoperative fasting insulin level (Fins); ➆: insulin resistance [measured by postoperative homeostasis model assessment-insulin resistance (HOMA-IR)]; ➇: the serum levels of C-reactive protein (CRP) within the first 24 h after surgery; ➈: postoperative scores of thirst; ➉: postoperative scores of hunger; ⑪: postoperative scores of anxiety; ⑫: postoperative scores of nausea and vomit; ⑬: postoperative scores of fatigue; ⑭: postoperative scores of weakness; ⑮ the occurrence of postoperative infection. *: 2 h before the surgery; †: 400 mL—between 9:00 and 11:00 p.m. before the surgery, and 400 mL—2–3 h before the surgery; ‡: 800 mL—8 h before the surgery, and 400 mL—2 h before the surgery; § : 1000 ml—8 h before the surgery and 500 mL—2 h before the surgery; ¶ : 400 mL in the evening before surgery and 400 mL in the morning on the day of surgery; **: 400 mL—8 h before the surgery and 200 mL—2 h before the surgery; ††: 474 mL —at the evening drinking and 237 mL — 3 h before the operation; §§ : 3 h before the surgery; ¶¶ : 4 h before the surgery; ***: 600 mL—8:00 p.m. before the surgery and 300 mL—2–3 h before the surgery; †††: oral from 8 PM before the operation and stop consumption 2 h before the planned time of operation; ‡‡‡: 250 ml- given 15, 11, and 4 h before surgery; §§§ : 710 mL —in the evening and 355 mL—2 h before surgery; ¶¶¶ : 500 mL—between 9:00 and 11:00 p.m. before the surgery, and 500 mL—2 h before the surgery. #: Procedures included otorhinolaryngological surgery, orthopedic/plastic surgery, gynecological surgery, breast and thyroid surgery, or thoracic surgery. ^: Open or laparoscopic. ##: Procedures included subtotal gastrectomy, hemicolectomy, and anterior resection. ^^: Procedures included laparoscopic Roux-en-Y gastric bypass, Laparoscopic sleeve gastrectomy. ###: Procedures included CABG and spinal surgical; ^^^: Procedures included total abdominal hysterectomy bilateral salpingo-oophorectomy, salpingo-oophorectomy, radical hysterectomy, and debulking tumor; N.S, not stated; ASA, American Society of Anesthesiologists; VAS, visual analog scale; CABG, coronary artery bypass grafting; iv, intravenous perfusion.

### Risk of bias and quality of evidence

**FIGURE 2:**
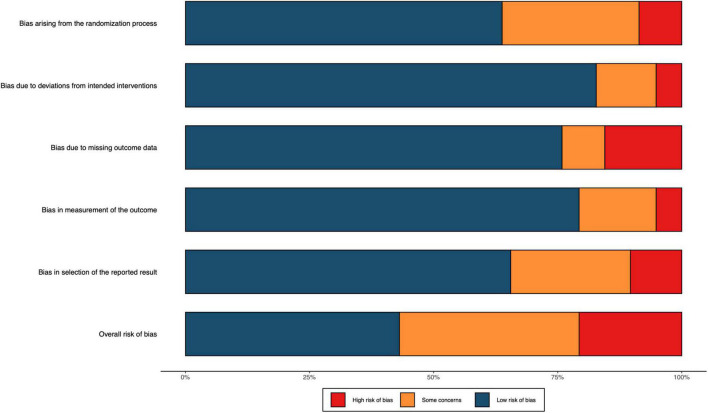
Risk of bias included RCTs. The colors in the bar next to each row/criteria represent the percentage of total studies falling within the high risk of bias/some concerns/low risk of bias.

The overall quality of RCTs included in the network was high and moderate. The risk of bias of 58 studies included in the meta-analysis is shown in Figure 2 (details of the risk of bias 2.0 assessment in each trial are shown in Supplementary Figure 1). According to the risk of bias 2.0 tool of Cochrane Collaboration, 25 (43%) studies were high-quality across all domains and 12 RCTs (21%) were at high risk of bias.

### Primary outcome

**FIGURE 3:**
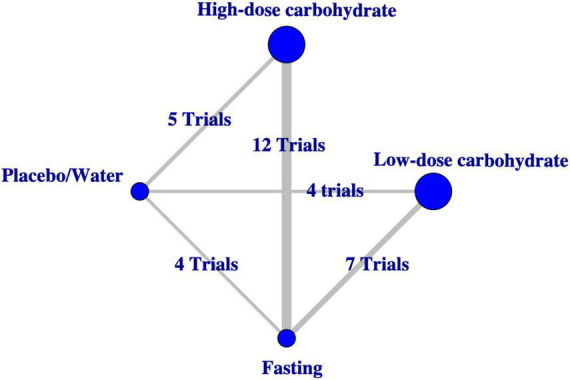
Network plot of evidence comparing different interventions for the primary outcome.

The primary outcome of this study was postoperative insulin resistance, and it was measured by the homeostasis model assessment-insulin resistance (HOMA-IR) method. The network plot for the primary outcome is shown in Figure 3. Each circle represented an intervention, and the area of each circle was proportional to the number of patients for which the intervention was accepted and indicated the sample size, and the width of the line was proportional to the number of trials that directly compared the two interventions.

**FIGURE 4:**
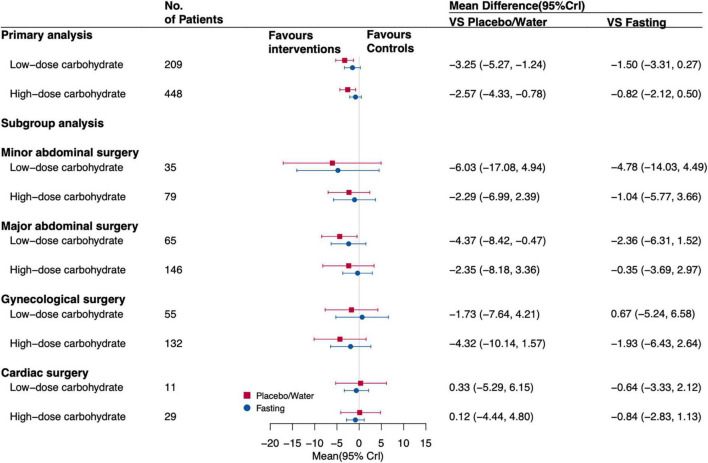
Forest plot for the estimates of different interventions on IR of postoperative patients. Values are mean differences (MDs) with 95% confidence intervals (Crls).

Twenty-four studies with 1,390 patients reported insulin resistance during the study period (27–51). Both interventions associated insulin resistance compared with placebo/water, with MD ranging from –3.25 (95%CrI, –5.27 to –1.24) for administrated oral low-dose carbohydrate to –2.57 (95%CrI, –4.33 to –0.78) for oral high-dose carbohydrate loading before surgery. The subgroup analysis based on the category of surgery revealed that the association of oral low-dose carbohydrate compared to placebo/water would correlate with insulin resistance (MD, –4.37 [95%CrI, –8.42 to –0.47]) for patients undergoing major abdominal surgery. Figure 4 shows the results. The result of CineMA represents the confidence in this estimate was low (Supplementary Table 2).

**FIGURE 5:**
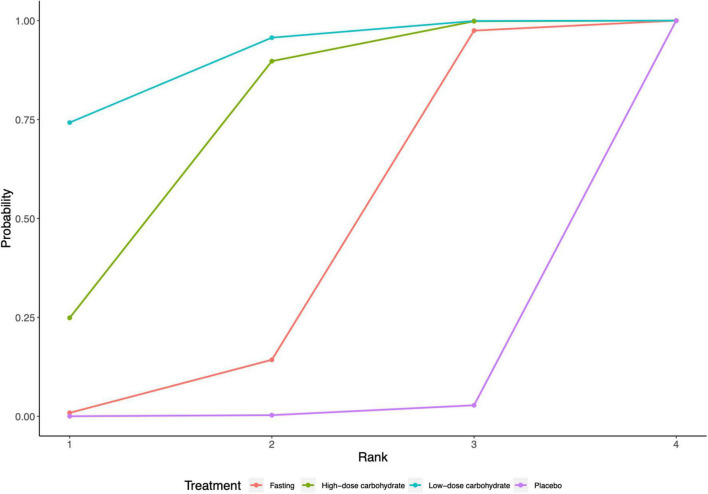
Surface under the cumulative ranking curve (SUCRA) for HOMA-IR.

Among all trials included, oral low-dose carbohydrate loading had the highest probability of being the best intervention (SUCRA value of 0.74 compared with other interventions). The corresponding results of SUCRA values are shown in Figure 5. Inconsistency analysis calculated by the node split method showed no significant difference between direct and indirect evidence of this network model, with *P*-value ranging from 0.05 to 0.32 (Supplementary Table 3). The result of the network meta-regression shows that the covariates we included may not affect the value of insulin resistance (Supplementary Table 4).

**FIGURE 6:**
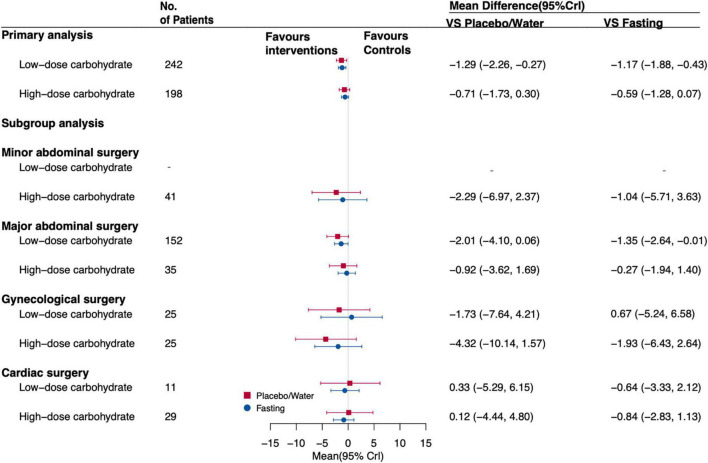
Forest plot for the estimates of different interventions on IR of postoperative patients that excluded trials at high risk of bias and data for the imputation methods. Values are mean differences (MDs) with 95% confidence intervals (CrIs).

After excluding studies with a high risk of bias and data of trials with imputation methods (network plot is shown in Supplementary Figure 2), there was an association of oral low-dose carbohydrate loading (MD, –1.29 [95%CrI, –2.26 to –0.27]) with insulin resistance for postoperative patients compared with placebo/water remained. Oral low-dose carbohydrate loading (MD, –1.17 [95% CrI, –1.88 to –0.43]) administration was associated with insulin resistance compared with fasting. The subgroup analysis showed that when patients undergoing major abdominal surgery, administrated oral low-dose carbohydrates before surgery was associated with insulin resistance (MD, –1.35 [95% CrI, –2.64 to –0.01]) compared with fasting. Figure 6 shows the forest plot results. And the SUCRA followed a similar pattern, with oral low-dose carbohydrates having the highest probability of being the best intervention when compared with other interventions; the SUCRA value is 0.88 (Supplementary Table 5).

A comparison-adjusted funnel plot for postoperative insulin resistance implies the presence of publication bias between the carbohydrate groups and controls (Supplementary Figure 3).

### Secondary outcomes

Supplementary Figure 4 represents network plots for each secondary outcome. The confidence in these estimates was generally moderate to very low (Supplementary Table 6).

#### Residual gastric volume during the surgery (mL)

Four studies reported RGV of intraoperative, involving 1,062 participants (52–55). The multiple-treatments meta-analysis results are shown in Table 2. There was no statistically significant difference between the groups in the network.

**TABLE 2:** Network meta-analysis matrix of secondary outcomes.

Outcomes	Treatment estimates are MDs/ORs and 95% Crls of the column-defining intervention compared with the row-defining intervention for different outcomes				
Residual gastric volume during the surgery ¶ (mL)	Low-dose carbohydrate				
	–2.51 (–16.31, 11.61)	High-dose carbohydrate			
	–	–	Carbohydrate, iv^		
	–0.81 (–14.99, 13.18)	1.66 (–6.74, 9.82)	–	Placebo/Water	
	–2.39 (–9.71, 4.99)	0.07 (–12.04, 12.11)	–	–1.62 (–13.76, 10.96)	Fasting
Postoperative insulin sensitivity ¶ (mg/kg/min)	Low-dose carbohydrate				
	0.28 (–1.62, 2.14)	High-dose carbohydrate			
	–0.75 (–3.55, 2.06)	–1.02 (–3.15, 1.13)	Carbohydrate, iv		
	0.30 (–1.49, 2.09)	0.02 (–0.56, 0.66)	1.05 (–1.08, 3.20)	Placebo/Water	
	0.45 (–1.65, 2.52)	0.16 (–0.91, 1.28)	1.20 (–0.66, 3.05)	0.15 (–0.96, 1.23)	Fasting
Postoperative fasting plasma glucose ¶ (mmol/L)	Low-dose carbohydrate				
	–0.28 (–0.86, 0.3)	High-dose carbohydrate			
	–0.81 (–1.67, 0.07)	–0.53 (–1.33, 0.29)	Carbohydrate, iv		
	–0.11 (–0.67, 0.47)	0.17 (–0.25, 0.60)	0.70 (–0.12, 1.52)	Placebo/Water	
	–0.28 (–0.78, 0.23)	0.00 (–0.38, 0.37)	0.53 (–0.27, 1.32)	–0.17 (–0.62, 0.27)	Fasting
Postoperative fasting insulin level ¶ (μU/mL)	Low-dose carbohydrate				
	–0.12 (–6.98, 6.99)	High-dose carbohydrate			
	–18.67 (–34.96, –2.31)	–18.58 (–34.29, –2.99)	Carbohydrate, iv^		
	–5.65 (–12.39, 1.21)	–5.53 (–10.61, –0.62)	13.03 (–1.79, 27.85)	Placebo/Water	
	–3.34 (–9.44, 2.75)	–3.23 (–7.96, 1.34)	15.35 (–0.54, 31.08)	2.31 (–3.32, 7.87)	Fasting
The serum levels of C-reactive protein within the first 24 h after surgery ¶ (mg/L)	Low-dose carbohydrate				
	7.12 (–30.65, 46.93)	High-dose carbohydrate			
	–	–	Carbohydrate, iv^		
	5.83 (–31.11, 45.84)	–1.42 (–28.28, 27.30)	–	Placebo/Water	
	–14.25 (–50.60, 22.64)	–21.28 (–46.71, 1.84)	–	–19.88 (–56.13, 12.37)	Fasting
Postoperative scores of pain¶	Low-dose carbohydrate				
	–0.35 (–5.33, 4.63)	High-dose carbohydrate			
	–1.26 (–6.83, 4.26)	–0.91 (–6.88, 5.02)	Carbohydrate, iv^		
	–0.77 (–5.53, 4.02)	–0.41 (–5.20, 4.34)	0.50 (–6.08, 7.13)	Placebo/Water	
	–1.52 (–6.32, 3.25)	–1.16 (–3.68, 1.29)	–0.25 (–5.84, 5.34)	–0.75 (–5.75, 4.22)	Fasting
Postoperative scores of patients’ satisfaction¶	Low-dose carbohydrate				
	1.26 (–6.00, 8.49)	High-dose carbohydrate			
	–	–	Carbohydrate, iv^		
	5.25 (–2.00, 12.50)	4.00 (–1.02, 9.01)	–	Placebo/Water	
	3.26 (–1.95, 8.46)	2.00 (–2.99, 7.04)	–	–2.00 (–7.00, 3.03)	Fasting
Postoperative scores of thirst¶	Low-dose carbohydrate				
	–1.49 (–12.63, 9.56)	High-dose carbohydrate			
	–	–	Carbohydrate, iv^		
	–0.90 (–9.14, 7.43)	0.59 (–6.78, 8.04)	–	Placebo/Water	
	–3.35 (–14.46, 7.68)	–1.87 (–5.61, 1.85)	–	–2.48 (–9.92, 4.92)	Fasting
Postoperative scores of hungry¶	Low-dose carbohydrate				
	–1.12 (–11.51, 9.34)	High-dose carbohydrate			
	–	–	Carbohydrate, iv^		
	–0.69 (–8.46, 7.07)	0.43 (–6.52, 7.35)	–	Placebo/Water	
	–2.24 (–12.64, 8.2)	–1.13 (–4.64, 2.34)	–	–1.57 (–8.50, 5.37)	Fasting
Postoperative scores of anxiety¶	Low-dose carbohydrate				
	0.20 (–11.76, 12.13)	High-dose carbohydrate			
	–	–	Carbohydrate, iv^		
	0.09 (–8.59, 8.80)	–0.11 (–8.25, 8.02)	–	Placebo/Water	
	–2.52 (–14.48, 9.46)	–2.72 (–8.88, 3.45)	–	–2.61 (–10.74, 5.56)	Fasting
Postoperative scores of nausea and vomit¶	Low-dose carbohydrate				
	–1.01 (–3.23, 1.22)	High-dose carbohydrate			
	–0.26 (–2.04, 1.51)	0.75 (–1.54, 3.04)	Carbohydrate, iv		
	–1.78 (–4.12, 0.53)	–0.76 (–1.76, 0.16)	–1.52 (–3.92, 0.84)	Placebo/Water	
	–1.36 (–3.43, 0.72)	–0.35 (–1.16, 0.46)	–1.10 (–3.24, 1.05)	0.42 (–0.60, 1.5)	Fasting
Postoperative scores of fatigue¶	Low-dose carbohydrate				
	–0.70 (–4.96, 3.57)	High-dose carbohydrate			
	–	–	Carbohydrate, iv^		
	–0.70 (–3.65, 2.26)	0.00 (–3.06, 3.08)	–	Placebo/Water	
	–1.49 (–6.53, 3.12)	–0.81 (–3.23, 1.25)	–	–0.81 (–4.81, 2.80)	Fasting
Postoperative scores of weakness¶	Low-dose carbohydrate^				
	–	High-dose carbohydrate			
	–	–	Carbohydrate, iv^		
	–	0.68 (–0.69, 2.12)	–	Placebo/Water	
	–	0.37 (–0.56, 1.47)	–	–0.31 (–1.67, 1.13)	Fasting
Occurrences of Postoperative infection#	Low-dose carbohydrate				
	0.63 (0.21, 2.00)	High-dose carbohydrate			
	–	–	Carbohydrate, iv^		
	–0.54 (–1.78, 0.66)	0.93 (0.42,1.70)	–	Placebo/Water	
	**0.42** **(0.20,0.81)**	0.71 (0.37,1.30)	–	0.72 (0.37,1.40)	Fasting

Postoperative insulin sensitivity: measured by hyperinsulinemic glucose clamp; comparisons between treatments read from left to right: a network estimate less than 0 (continuous variables) or 1 (dichotomous variables) indicates that the treatment reported in the column is more effective than the corresponding treatment reported in row. ¶ : Mean difference (MD) and 95% confidence intervals (Crls); #: odds ratios (ORs) and 95% confidence intervals (Crls); ^: No data available for this outcome. Significant results are in bold. Low-dose carbohydrate: The dose of oral carbohydrate is between 10 and 50 g before surgery (10–50 g); High-dose carbohydrate: The dose of oral carbohydrate is greater than 50 g before surgery (>50 g); Carbohydrate, iv: preoperative carbohydrate by intravenous perfusion; Placebo/Water: flavored sweetened drink/purified water; fasting: overnight fasting before the day of surgery.

#### Postoperative insulin sensitivity (mg/kg/min)

Seven trials measured insulin sensitivity by hyperinsulinaemic–euglycaemic clamp method, involving 170 participants. The results showed carbohydrate loading dose had no significant differences in any of the comparisons (Table 2).

#### Postoperative fasting plasma glucose (mmol/L)

Twenty-seven trials reported the FPG of patients after surgery, involving 1886 participants (30–37, 40–50, 56–65). Compared with the control groups, preoperative carbohydrate loading had no significant effect on postoperative FPG. Table 2 shows the results.

#### Postoperative Fin level (μU/mL)

Twenty-two studies were included, with data available for 1,379 participants (9, 29, 30, 33, 34, 36–46, 48–50, 61, 64, 65). Compared with placebo or water, high-dose carbohydrate loading before surgery was associated with a decrease in Fin level (MD, –5.53 [95%Crl, –10.61 to –0.62]). However, because the confidence interval was wide and close to insignificance, the results should be interpreted with caution. Table 2 displays the results of the multiple-treatments meta-analysis.

#### The serum levels of C-reactive protein within the first 24 h after surgery

Seven studies collected blood samples to assess the serum levels of CRP, with data available for 443 participants (9, 37, 38, 41, 44, 53, 66). Multiple-treatments meta-analysis shows no significant difference in any of the companions (Table 2).

#### Postoperative scores of pain

Eight studies reported postoperative scores of pain scores using a VAS, with data available on 739 participants (29, 35, 44, 62, 67–70). The results found no statistically significant difference after surgery (Table 2).

#### Postoperative scores of patients’ satisfaction

This was reported by two studies using a VAS, with data available on 140 participants (52, 57). Multiple-treatments meta-analysis found no significant difference in any of the treatments within the network (Table 2).

#### Postoperative scores of thirst

Six studies reported postoperative thirst scores using a VAS, with data available on 539 participants (35, 44, 57, 68, 69, 71). The results found no statistically significant difference after surgery (Table 2).

#### Postoperative scores of hungry

This was reported by six studies using a VAS, with data available on 539 participants (35, 44, 57, 68, 69, 71). Multiple-treatments meta-analysis found no significant difference in any of the treatments within the network (Table 2).

#### Postoperative scores of anxiety

Three studies reported postoperative anxiety scores; all trials used a VAS, with data available on 318 participants (35, 69, 71). The results found no statistically significant difference after surgery (Table 2).

#### Postoperative scores of nausea and vomit

Seven studies reported postoperative nausea and vomiting scores; all trials used a VAS, and data on 527 participants were available (40, 46, 57, 62, 68, 71, 72). Multiple-treatments meta-analysis found no significant difference in any of the treatments within the network (Table 2).

#### Postoperative scores of fatigue

This was reported by four studies using a VAS, with data available on 449 participants (57, 67–69). Multiple-treatments meta-analysis found no significant difference in any of the treatments within the network (Table 2).

#### Postoperative scores of weakness

Two studies reported postoperative weakness scores using a VAS, with data available on 126 participants (44, 71). The results found no statistically significant difference after surgery (Table 2).

#### The occurrences of postoperative infection

Eleven studies reported the occurrences of postoperative infection, with data available on 1,765 participants (36, 45, 49, 50, 54, 58, 59, 66, 73–75) (Table 2). The NMA result revealed that compared with fasting, low-dose carbohydrate could reduce the occurrences of postoperative infection with statistical significance (odds ratio, 0.42 [95%Crl: 0.20–0.81]). The results of the network meta-regression shows that the covariates we included may not affect the value of secondary outcomes, except the postoperative FPG (Supplementary Table 7).

The value of SUCRA represented that oral low-dose carbohydrate loading had the highest probability of being the best intervention relative to other interventions in patients’ postoperative comfort except for postoperative insulin sensitivity (mg/kg/min), fasting insulin levels (μU/mL), postoperative satisfaction, and weakness (Supplementary Table 8).

Network meta-regression showed that the covariates did not, indeed, influence the value of primary and secondary outcomes (Supplementary Table 9). When trials with a high risk of bias and imputed data were excluded, the results for the secondary outcomes were similar (Supplementary Table 11).

## Sensitivity analyses

A summary of clinical and statistical sensitivity analyses is given in Supplementary Tables 10, 11. In the clinical sensitivity, after splitting the “water/placebo” group into two separate arms, postoperative insulin resistance reported a significant MD of –4.02 (95% CrI [–6.46, –1.63]) for low-dose carbohydrate vs. placebo, and MD of –3.65 (95% CrI [–6.24, –1.06]) for high-dose carbohydrate vs. placebo, and the sensitivity analyses were consistent with the main analysis of the secondary outcomes. In the statistical sensitivity analyses, when excluding trials at high risk of bias and data for the imputation methods, oral low-dose carbohydrate loading compared to placebo/water associated with postoperative insulin resistance (MD, –1.29 [95% CrI, –2.26 to –0.27]) for postoperative patients, and compared with fasting, insulin resistance was correlated with oral low-dose carbohydrate (MD, –1.17 [95% CrI, –1.88 to –0.43]). The other results did not differ significantly.

## Discussion

### Summary of findings

The latest practice guidelines for preoperative fasting recommend that clear liquids may be ingested for up 2 h before an operation; however, it reported less thirst and hunger for fasting time of 2–4 h compared to more than 4 h of fasting, however, it reported equivocal findings for RGV, blood glucose values, hunger, and thirst of nutritional or carbohydrate drinks at 2–4 h relative to more than 4 h of fasting (1).

This NMA represents the most comprehensive analysis of currently available data regarding preoperative carbohydrate loading for patients undergoing elective surgery. We combined direct and indirect evidence from 58 trials comparing four different intervention arms in 4,936 patients undergoing elective surgery. The study that included sufficient numbers of patients to prove a potential association in clinical outcomes was of patients undergoing elective surgery, and it included the most patients available in the current literature. To maintain the homogeneity of interventions, our research divided the dose of carbohydrate loading into low dose (10–50 g) and high dose (>50 g). Our main findings indicate that among patients undergoing elective surgery, preoperative low-dose carbohydrate loading has been found to be associated with insulin resistance and postoperative infection rates.

Three published meta-analyses explored the influence of low-carbohydrate loading on postoperative outcomes (2, 8, 12). However, reports of the effects of carbohydrate loading on insulin sensitivity remain inconsistent. Smith et al. (8) conducted that no significant association was between carbohydrate loading and insulin resistance An earlier NMA of 43 RCTs found that only high-dose carbohydrate administration resulted in a statistically significant associated with insulin resistance compared with fasting, and water or placebo, but with wide confidence intervals so the results are not credible (12). A recent meta-analysis has investigated that compared with fasting, preoperative administration of carbohydrate associated with insulin resistance (2). In our study, we found that oral carbohydrate loading was associated with insulin resistance compared with placebo or water, and the association was still observed in an analysis of excluded high-risk trials and data for the imputation methods. A separate subgroup analysis based on the surgical categories identified the true effect of low-dose carbohydrate loading on insulin resistance, especially those undergoing major abdominal surgery that would otherwise be confounded by other surgical categories. This effect might be due to the preoperative carbohydrate loading, which stimulates an endogenous insulin release and switches off the overnight fasting metabolic state, toward anabolism (63). It should be mentioned that the confidence effect estimate is low or very low, and the significant heterogeneity among studies (different categories of surgery, different types of carbohydrates, and different populations); therefore, the result regarding the effect of carbohydrate loading on insulin resistance must be interpreted with caution.

The present meta-analysis found that oral high-dose carbohydrate (>50 g) was more effective in postoperative outcomes than relative to low-dose carbohydrate, and there is no dose–response relationship between carbohydrate and postoperative outcomes. This may be related to the fact that there is less data available in the network for low-dose carbohydrate comparisons, so some results have wider confidence intervals than in high-dose comparisons.

The gold standard of insulin sensitivity is measured by the hyperinsulinemic–euglycemic clamp method in humans (76). However, we found a small number of studies (*n* = 7) for this outcome, which could be due to the fact that it is a time-consuming, labor-intensive, and invasive procedure. The multiple-treatments meta-analysis found no evidence that carbohydrate loading was more or less effective in reducing insulin sensitivity compared with placebo/water or fasting. Therefore, more randomized controlled trials need to be included in future analyses to further confirm this outcome.

A recent meta-analysis has investigated that compared with fasting, preoperative administration of carbohydrates decreased patients’ thirst, hungry, and pain (2). Meanwhile, in our study, there was no difference in postoperative patients’ comfort between the administration of preoperative carbohydrates and control groups, and no other significant differences were found in any of the other secondary outcomes. However, some of these results had wide confidence intervals, indicating that data availability is limited. Future well-designed randomized studies will need to examine the biochemical effects and recovery of preoperative carbohydrate loading in elective surgery.

### Strengths and limitations

This review has some strengths: First, a comprehensive search was conducted to identify eligible trials; independent study selection, data extraction, and risk of bias assessment were performed by two reviewers; and the CINeMA was used to assess confidence in the NMA results. Second, we also conducted a network meta-regression to evaluate which variables might influence the postoperative outcomes. This review used a Bayesian framework to overcame the tendency of the frequentist approach to be unstable in parameter estimation and obtain biased results (77). Third, we tested different model assumptions to verify the reliability of outcomes in this NMA. Fourth, a NMA is performed to analyze the effect of preoperative carbohydrate loading on various postoperative recovery indicators among elective surgery patients, compensating for the lack of direct comparison between them.

This study has several limitations. First, the results of this meta-analysis are highly dependent on the quality of the trials included. According to the CINeMA results, the evaluation of the credibility of results was from moderate to very low, and there was large uncertainty regarding all the estimates. Second, although 58 RCTs were retrieved, only 21 trials reported postoperative low-dose carbohydrate administration in the network, two studies reported preoperative carbohydrate by intravenous perfusion, and there were relatively few direct comparisons. Third, this may, however, be a type II error (false-negative findings), as only a few trials are available to assess postoperative outcome indicators in many second outcomes. Fourth, small trials tend to report larger beneficial effects than large trials; however, only three trials in our review included more than 100 patients per arm, which may introduce bias due to small-study effects (78). Fifth, the SUCRA value was used to estimate a ranking probability of comparative effectiveness between the different interventions. Sixth, many trials, lack good design, resulting in combining different types of carbohydrates into one group and placebo and water into one group for the main analysis. Finally, double-blinding was not applied in many trials designs included, which may affect the results, but this is also difficult to resolve because fasting and drinking are easily known by the participants, and subsequent experiments need to be further refined.

## Conclusion

In summary, when compared with fasting and placebo/water, preoperative carbohydrate appears to be associated with some postoperative outcomes; however, more research into these drinks, preferably multi-types carbohydrate trials are required to improve the strength of the evidence and inform clinical practice.

## Data availability statement

The original contributions presented in this study are included in the article/Supplementary material, further inquiries can be directed to the corresponding author.

## Author contributions

ET, YC, YR, and YYZ designed and conducted the research. ET completed the first draft of the manuscript. YZ, SS, and SQ analyzed the data and performed the statistical analyses. CD, YH, and LY substantively revised it. XT critically reviewed the manuscript. All authors contributed to the design of the research (project conception, development of the overall research plan) and approved the final manuscript.
