# Supplementary material for: Effects of preoperative carbohydrate loading on recovery after elective surgery: A systematic review and Bayesian network meta-analysis of randomized controlled trials
Source: Front Nutr. 2022 Nov 23;9:951676. doi: 10.3389/fnut.2022.951676 (PMC9726728; doi:10.3389/fnut.2022.951676)
Supplement: Supplementary file 1 [file Data_Sheet_1.docx]

*Supplement materials*

1.Supplementary Tables

1.1 Supplementary Table S1. Search strategy for electronic databases

| **Database** | **Search Terms** |
| --- | --- |
| **MEDLINE(PubMed)** | (((((("Fasting"[Mesh]) OR ((((((((((((fasting) OR (starving)) OR (drink*)) OR (eat*)) OR (meal)) OR (meals)) OR (food)) OR (feed)) OR (solids)) OR (intake*)) OR (liquid*)) OR (fluid*))) OR ("Water"[Mesh])) OR (((NPO) OR (non per os)) OR (nil per os))) OR (diet restriction)) AND (("Preoperative Period"[Mesh]) OR ((((((((((((((((((((surgery*) OR (operation*)) OR (operation care)) OR (operative intervention)) OR (operative surgical procedure)) OR (operative treatment)) OR (research surgery)) OR (resection)) OR (surgery, operative)) OR (surgical care)) OR (surgical correction)) OR (surgical operation)) OR (surgical practice)) OR (surgical procedures, operative)) OR (surgical repair)) OR (surgical research)) OR (surgical restoration)) OR (surgical service)) OR (surgical therapy)) OR (surgical treatment)))) AND((((((((prediabetic state) OR (prediabetes)) OR (diabetes mellitus, type 2[MeSH Terms])) OR (diabetes mellitus, type 2)) OR (type 2 diabetes mellitus)) OR ((((insulin) AND (resistance)) AND (insulin resistance[MeSH Terms])) OR (insulin resistance))) OR (((insulin) AND (sensitivity)) OR (insulin sensitivity))) OR ((blood glucose) OR ((glucose) AND (blood)) OR (glucose, blood))) Filters: Randomized Controlled Trial, Humans. |
| **Database** | **Search Terms** |
| **Web of Science** | #1:TS=( Fasting OR starving OR drink* OR eat* OR meal OR meals OR food OR feed OR solids OR intake* OR liquid* OR fluid* OR Water OR NPO OR non per os OR nil per os OR diet restriction)  #2:TS=(Preoperative Period OR surgery* OR operation* OR operation care OR operative intervention OR operative surgical procedure OR operative treatment OR research surgery OR resection OR surgery,operative OR surgical care OR surgical correction OR surgical operation OR surgical practice OR surgical procedures,operative OR surgical repair OR surgical research OR surgical restoration OR surgical service OR surgical therapy OR surgical treatment)  # 3:TS= ((((((((prediabetic state) OR (prediabetes) ) OR (diabetes mellitus, type 2) ) OR (diabetes mellitus, type 2) ) OR (type 2 diabetes mellitus) ) OR ((((insulin) AND (resistance) ) AND (insulin resistance) ) OR (insulin resistance) )) OR (((insulin) AND (sensitivity) ) OR (insulin sensitivity) )) OR ((blood glucose) OR ((glucose) AND (blood) ) OR (glucose, blood) ))  # 4:#3 AND #2 AND #1 |
| **EMBASE** | ('surgery'/exp OR 'diagnosis, surgical' OR 'diagnostic techniques, surgical' OR 'operation' OR 'operation care' OR 'operative intervention' OR 'operative repair' OR 'operative restoration' OR 'operative surgery' OR 'operative surgical procedure' OR 'operative surgical procedures' OR 'operative treatment' OR 'research surgery' OR 'resection' OR 'specialties, surgical' OR 'surgery' OR 'surgery, operative' OR 'surgical care' OR 'surgical correction' OR 'surgical diagnosis' OR 'surgical diagnostic techniques' OR 'surgical exposure' OR 'surgical intervention' OR 'surgical management' OR 'surgical operation' OR 'surgical practice' OR 'surgical procedures, operative' OR 'surgical repair' OR 'surgical research' OR 'surgical restoration' OR 'surgical service' OR 'surgical specialty' OR 'surgical specialties' OR 'surgical specialty' OR 'surgical therapy' OR 'surgical treatment' OR 'selective operation') AND 'blood glucose' AND ('insulin resistance'/exp OR 'insulin sensitivity'/exp OR 'insulin insensitivity' OR 'insulin sensitivity' OR 'insulin sensitivity test' OR 'insulin test' OR 'sensitivity, insulin' OR 'prediabetic state' OR prediabetes OR 'diabetes mellitus, type 2' OR 'type 2 diabetes mellitus') AND [humans]/lim |
| **Database** | **Search Terms** |
| **EBSCO** | TX ( (fasting OR starving OR drink* OR eat* OR meal OR meals OR food OR feed OR solids OR intake* OR liquid* OR fluid* OR Water OR NPO OR non per os OR nil per os OR diet restriction) ) AND TX ( (Preoperative Period OR surgery* OR operation* OR operation care OR operative intervention OR operative surgical procedure OR operative treatment OR research surgery OR resection OR surgery, operative OR surgical care OR surgical correction OR surgical operation OR surgical practice OR surgical procedures, operative OR surgical repair OR surgical research OR surgical restoration OR surgical service OR surgical therapy OR surgical treatment) ) AND TX ( ((((((((prediabetic state) OR (prediabetes) ) OR (diabetes mellitus, type 2) ) OR (diabetes mellitus, type 2) ) OR (type 2 diabetes mellitus) ) OR ((((insulin) AND (resistance) ) AND (insulin resistance) ) OR (insulin resistance) )) OR (((insulin) AND (sensitivity) ) OR (insulin sensitivity) )) OR ((blood glucose) OR ((glucose) AND (blood) ) OR (glucose, blood)))) |
| the Cochrane Central Register of Controlled Trials | #1 MeSH descriptor: [Fasting] explode all trees  #2 fasting  #3 starving  #4 drink*  #5 eat*  #6 meal  #7 meals  #8 food  #9 solids  #10 oral NEXT (intake* or liquid* or fluid*)  #11 MeSH descriptor: [Water Deprivation] explode all trees  #12 (fluid or water) NEXT deprivation  #13 nil by mouth  #14 nulla per os  #15 NPO  #16 preoperative fasting  #17 diet restriction  #18 #1 OR #2 OR #3 OR #4 OR #5 OR #6 OR #7 OR #8 OR #9 OR #10 OR #11 OR #12 OR #13 OR #14 OR #15 OR #16 OR #17  #19 MeSH descriptor: [General Surgery] explode all trees  #20 operation*  #21 operation care  #22 operative intervention  #23 operative surgical procedure  #24 operative treatment  #25 research surgery  #26 resection  #27 surgery, operative  #28 surgical care  #29 surgical correction  #30 surgical exposure  #31 surgical intervention  #32 surgical management  #33 surgical operation  #34 surgical practice  #35 surgical procedures, operative  #36 surgical research  #37 surgical restoration  #38 surgical service  #39 surgical therapy  #40 surgical treatment  #41 #20 OR #21 OR #22 OR #23 OR #24 OR #25 OR #26 OR #27 OR #28 OR #29 OR #30 OR #31 OR #32 OR #33 OR #34 OR #35 OR #36 OR #37 OR #38 OR #39 OR #40  #42 #18 AND #41  #43 MeSH descriptor: [Insulin Resistance] explode all trees  #44 prediabetic state  #45 diabetes mellitus, type 2  #46 type 2 diabetes mellitus  #47 insulin sensitivity  #48 blood glucose  #49 #42 OR #43 OR #44 OR #45 OR #46 OR #47 OR #48  #50 #42 AND #49 |

**1.2 Supplementary Table S2.** Confidence effect estimates for postoperative HOMA-IR outcome in different preoperative interventions

| **Outcomes** | **Comparison** | **Within-study bias** | **Reporting bias** | **Indirectness** | **Imprecision** | **Heterogeneity** | **Incoherence** | **Confidence rating** |
| --- | --- | --- | --- | --- | --- | --- | --- | --- |
| **HOMA-IR** | Low-dose carbohydrate: Placebo | Some concerns | undetected | No concerns | No concerns | No concerns | No concerns | Low |
|  | Low-dose carbohydrate: Fasting | Some concerns | undetected | No concerns | No concerns | Major concerns | No concerns | Low |
|  | High-dose carbohydrate: Placebo | Some concerns | undetected | No concerns | No concerns | Major concerns | No concerns | Low |
|  | High-dose carbohydrate: Fasting | Some concerns | undetected | No concerns | Major concerns | No concerns | No concerns | Low |

**1.3 Supplementary Table S3.** Network meta-analysis results for the effect of preoperative carbohydrate loading on recovery after elective surgery

| **Outcomes** |  | **Direct estimate**  **MD (95%Crl)** | **Indirect estimate**  **MD(95%Crl)** | **Network estimate**  **MD (95%Crl)** | **Incoherence**  **(P-value)** |
| --- | --- | --- | --- | --- | --- |
| HOMA-IR | Low-dose carbohydrate vs Placebo | -2.50  (-4.80,-0.19) | -4.40  (-7.80,-0.88) | **-3.02**  **(-4.80, -1.26)** | 0.35 |
|  | Low-dose carbohydrate vs Fasting | -1.60  (-3.20,-0.07) | -4.90  (-9.60,-0.33) | **-1.72**  **(-3.33, -0.18)** | 0.17 |
|  | High-dose carbohydrate vs Placebo | -2.60  (-4.60,-0.53) | -0.86  (-3.40,1.70) | **-1.90**  **(-3.43, -0.33)** | 0.28 |
|  | High-dose carbohydrate vs Fasting | -0.27  (-1.40,0.90) | -2.80  (-5.80,0.18) | -0.60  (-1.74, 0.54) | 0.11 |

HOMA-IR: the Homeostasis Model Assessment of Insulin Resistance; MD: mean difference.

1.4 Supplementary Table S4. Network meta-regression for different covariates

| **Outcome** |  |  | **50% (95%Crl)** |
| --- | --- | --- | --- |
| HOMA-IR | Adjusted for the risk of bias | Beta[1] | -2.88 (-5.43,2.80) |
|  |  | Beta[2] | -2.14 (-6.14,2.30) |
|  |  | Beta[3] | 0.87 (-2.11,4.08) |
|  |  | Beta[4] | -0.29 (-3.47,3.05) |
|  | Adjusted for blinding | Beta[1] | 1.27(-4.75,20.41) |
|  |  | Beta[2] | 1.15(-1.95,3.65) |
|  |  | Beta[3] | 3.82 (-0.54,9.98) |
|  |  | Beta[4] | 0.43(-4.30,4.88) |
|  | Adjusted for the type of surgery | Beta[1] | 0.77(-21.55,4.38) |
|  |  | Beta[2] | 0.93(-1.92,4.06) |
|  |  | Beta[3] | -0.83(-9.77,2.71) |
|  |  | Beta[4] | 0.47(-4.17,4.66) |

HOMA-IR: the Homeostasis Model Assessment of Insulin Resistance;

CrIs= Credible intervals;

[1] Low-dose carbohydrate versus Placebo/Water ; [2] Low-dose carbohydrate versus Fasting; [3] High-dose carbohydrate versus Placebo/Water;

[4] High-dose carbohydrate versus Fasting; [5] Carbohydrate, iv versus Placebo/water; [6] Carbohydrate, iv versus Fasting;

1.5 Supplementary Table S5. Cumulative ranking of interventions for different outcomes that excluded trials at high risk of bias and data for the imputation methods.

| **Outcome** | **Rank** | **Intervention** | **SUCRA** |
| --- | --- | --- | --- |
| HOMA-IR | 1 | Low-dose carbohydrate | 92.2% |
|  | 2 | High-dose carbohydrate | 6.7% |
|  | 3 | Fasting | 1.1% |
|  | 4 | Placebo | 0.06% |

HOMA-IR: the Homeostasis Model Assessment of Insulin Resistance;

SUCRA: the surface under the cumulative ranking

**1.6 Supplementary Table S6.** Confidence effect estimates for secondary outcomes in different preoperative interventions

| **Outcomes** | **Comparison** | **Within-study bias** | **Reporting bias** | **Indirectness** | **Imprecision** | **Heterogeneity** | **Incoherence** | **Confidence rating** |
| --- | --- | --- | --- | --- | --- | --- | --- | --- |
| **RGV** | Low-dose carbohydrate: Placebo | Some concerns | undetected | No concerns | Major concerns | No concerns | No concerns | Very low |
|  | Low-dose carbohydrate: Fasting | Major concerns | undetected | No concerns | Major concerns | No concerns | No concerns | Very low |
|  | High-dose carbohydrate: Placebo | No concerns | undetected | No concerns | Some concerns | No concerns | No concerns | Low |
|  | High-dose carbohydrate: Fasting | No concerns | undetected | No concerns | Some concerns | No concerns | No concerns | Low |
| Postoperative insulin sensitivity | Low-dose carbohydrate:  Carbohydrate, iv | No concerns | undetected | No concerns | Some concerns | No concerns | No concerns | Moderate |
|  | Low-dose carbohydrate: Placebo | No concerns | undetected | No concerns | Some concerns | No concerns | No concerns | Moderate |
|  | Low-dose carbohydrate:  Fasting | No concerns | undetected | No concerns | Some concerns | No concerns | No concerns | Moderate |
|  | High-dose carbohydrate:  Carbohydrate, iv | No concerns | undetected | No concerns | Some concerns | No concerns | Some concerns | Low |
|  | High-dose carbohydrate:  Placebo | No concerns | undetected | No concerns | No concerns | Some concerns | No concerns | Moderate |
|  | High-dose carbohydrate:  Fasting | No concerns | undetected | No concerns | Some concerns | No concerns | No concerns | Low |
|  | Carbohydrate, iv: Placebo | No concerns | undetected | No concerns | Some concerns | No concerns | No concerns | Moderate |
|  | Carbohydrate, iv: Fasting | Some concerns | undetected | No concerns | Some concerns | No concerns | Some concerns | Low |
| **Postoperative FPG** | Low-dose carbohydrate: Placebo | No concerns | undetected | No concerns | Some concerns | Major concerns | No concerns | Low |
|  | Low-dose carbohydrate: Fasting | Some concerns | undetected | No concerns | No concerns | Major concerns | No concerns | Very low |
|  | High-dose carbohydrate: Placebo | No concerns | undetected | No concerns | Some concerns | No concerns | No concerns | Moderate |
|  | High-dose carbohydrate: Fasting | Some concerns | undetected | No concerns | Some concerns | No concerns | No concerns | Low |
|  | Carbohydrate, iv: Placebo | Some concerns | undetected | No concerns | Some concerns | No concerns | No concerns | Low |
|  | Carbohydrate, iv: Fasting | Some concerns | undetected | Some concerns | Some concerns | No concerns | No concerns | Low |
| **Postoperative Fins** | Low-dose carbohydrate: Placebo | Some concerns | undetected | No concerns | No concerns | Some concerns | No concerns | Low |
|  | Low-dose carbohydrate: Fasting | No concerns | undetected | No concerns | No concerns | Some concerns | No concerns | Moderate |
|  | High-dose carbohydrate: Placebo | Some concerns | undetected | No concerns | No concerns | Some concerns | No concerns | Low |
|  | High-dose carbohydrate: Fasting | Some concerns | undetected | No concerns | Major concerns | No concerns | No concerns | Very low |
| **CRP** | Low-dose carbohydrate: Placebo | Some concerns | undetected | No concerns | Major concerns | No concerns | No concerns | Very low |
|  | Low-dose carbohydrate: Fasting | No concerns | undetected | No concerns | Major concerns | No concerns | No concerns | Very low |
|  | High-dose carbohydrate: Placebo | Some concerns | undetected | No concerns | Major concerns | No concerns | No concerns | Very low |
|  | High-dose carbohydrate: Fasting | Some concerns | undetected | No concerns | Major concerns | No concerns | No concerns | Very low |
| **Postoperative pain** | Low-dose carbohydrate: Placebo | Some concerns | undetected | No concerns | Major concerns | No concerns | No concerns | Very low |
|  | Low-dose carbohydrate: Fasting | Major concerns | undetected | No concerns | Major concerns | No concerns | No concerns | Very low |
|  | High-dose carbohydrate: Placebo | Some concerns | undetected | No concerns | Major concerns | No concerns | No concerns | Very low |
|  | High-dose carbohydrate: Fasting | Some concerns | undetected | No concerns | Major concerns | No concerns | No concerns | Very low |
|  | Carbohydrate, iv: Placebo | Some concerns | undetected | Some concerns | Major concerns | No concerns | No concerns | Very low |
|  | Carbohydrate, iv: Fasting | Major concerns | undetected | No concerns | Major concerns | No concerns | No concerns | Very low |
| **Postoperative patient satisfaction** | Low-dose carbohydrate: Placebo | undetected | undetected | undetected | undetected | undetected | undetected | undetected |
|  | Low-dose carbohydrate: Fasting | undetected | undetected | undetected | undetected | undetected | undetected | undetected |
|  | High-dose carbohydrate: Placebo | undetected | undetected | undetected | undetected | undetected | undetected | undetected |
|  | High-dose carbohydrate: Fasting | undetected | undetected | undetected | undetected | undetected | undetected | undetected |
| **Postoperative thirst** | Low-dose carbohydrate: Placebo | Some concerns | undetected | Some concerns | Some concerns | No concerns | No concerns | Low |
|  | Low-dose carbohydrate: Fasting | Some concerns | undetected | No concerns | Some concerns | No concerns | No concerns | Low |
|  | High-dose carbohydrate: Placebo | Some concerns | undetected | No concerns | Some concerns | No concerns | No concerns | Low |
|  | High-dose carbohydrate: Fasting | Some concerns | undetected | No concerns | Some concerns | No concerns | No concerns | Low |
| **Postoperative hungry** | Low-dose carbohydrate: Placebo | Some concerns | undetected | Some concerns | Some concerns | No concerns | No concerns | Very low |
|  | Low-dose carbohydrate: Fasting | Some concerns | undetected | No concerns | Some concerns | No concerns | No concerns | Low |
|  | High-dose carbohydrate: Placebo | Some concerns | undetected | No concerns | Some concerns | No concerns | No concerns | Low |
|  | High-dose carbohydrate: Fasting | Some concerns | undetected | No concerns | Some concerns | No concerns | No concerns | Low |
| **Postoperative anxiety** | Low-dose carbohydrate: Placebo | Some concerns | undetected | undetected | undetected | undetected | undetected | undetected |
|  | Low-dose carbohydrate: Fasting | Some concerns | undetected | undetected | undetected | undetected | undetected | undetected |
|  | High-dose carbohydrate: Placebo | Some concerns | undetected | undetected | undetected | undetected | undetected | undetected |
|  | High-dose carbohydrate: Fasting | Some concerns | undetected | undetected | undetected | undetected | undetected | undetected |
| **Postoperative nausea and vomit** | Low-dose carbohydrate: Placebo | Major concerns | undetected | No concerns | No concerns | Major concerns | No concerns | Very low |
|  | Low-dose carbohydrate: Fasting | Major concerns | undetected | No concerns | Some concerns | No concerns | No concerns | Very low |
|  | High-dose carbohydrate: Placebo | Some concerns | undetected | No concerns | No concerns | Major concerns | No concerns | Very low |
|  | High-dose carbohydrate: Fasting | Some concerns | undetected | No concerns | Some concerns | No concerns | No concerns | Low |
|  | Carbohydrate, iv: Placebo | Major concerns | undetected | No concerns | Some concerns | No concerns | No concerns | Very low |
|  | Carbohydrate, iv: Fasting | Major concerns | undetected | No concerns | Some concerns | No concerns | No concerns | Very low |
| **Postoperative fatigue** | Low-dose carbohydrate: Placebo | undetected | undetected | undetected | undetected | undetected | undetected | undetected |
|  | Low-dose carbohydrate: Fasting | undetected | undetected | undetected | undetected | undetected | undetected | undetected |
|  | High-dose carbohydrate: Placebo | undetected | undetected | undetected | undetected | undetected | undetected | undetected |
|  | High-dose carbohydrate: Fasting | undetected | undetected | undetected | undetected | undetected | undetected | undetected |
| **Postoperative weakness** | High-dose carbohydrate: Placebo | Some concerns | undetected | No concerns | undetected | undetected | undetected | undetected |
|  | High-dose carbohydrate: Fasting | Some concerns | undetected | No concerns | undetected | undetected | undetected | undetected |
| **Postoperative infection** | Low-dose carbohydrate: Placebo | Some concerns | undetected | Some concerns | Major concerns | No concerns | No concerns | Very low |
|  | Low-dose carbohydrate: Fasting | No concerns | undetected | No concerns | No concerns | Some concerns | No concerns | Moderate |
|  | High-dose carbohydrate: Placebo | No concerns | undetected | No concerns | Major concerns | No concerns | No concerns | Very low |
|  | High-dose carbohydrate: Fasting | Some concerns | undetected | No concerns | Major concerns | No concerns | No concerns | Very low |

RGV: residual gastric volume; FPG: fasting plasma glucose; Fins: fasting serum insulin; CRP: C-reactive protein.

**1.7 Supplementary Table S7** Network meta-analysis secondary results for the effect of preoperative carbohydrate loading on recovery after elective surgery

| **Outcomes** |  | **Direct estimate MD/OR**  **(95%Crl)** | **Indirect estimate**  **MD/OR**  **(95%Crl)** | **Network estimate**  **MD/OR**  **(95%Crl)** | **Incoherence**  **(P-value)** |
| --- | --- | --- | --- | --- | --- |
| RGV during the surgery(ml) | Low-dose carbohydrate vs Placebo | -5.04  (-50.00,-49.50) | -2.83  (-12.3,7.64) | -2.81  (-11.93, 7.54) | 0.93 |
|  | Low-dose carbohydrate vs Fasting | -2.34  (-10.12,5.29) | 0.28  (-47.81,-52.03) | -2.41  (-9.99, 5.31) | 0.91 |
|  | High-dose carbohydrate vs Placebo | -2.01  (-8.28,4.26) | 0.05  (-51.83,51.98) | -2.02  (-6.35, 4.84) | 0.94 |
|  | High-dose carbohydrate vs Fasting | 1.00  (-5.32,7.36) | -3.99  (-53.94,46.95) | -1.61  (-7.20, 5.44) | 0.84 |
| Postoperative insulin sensitivity | Low-dose carbohydrate vs Carbohydrate, iv | NA | NA | -0.75  (-3.55, 2.06) | NA |
|  | Low-dose carbohydrate vs Placebo | 0.31  (-1.50,2.10) | NA | 0.30  (-1.49, 2.09) | NA |
|  | Low-dose carbohydrate vs Fasting | NA | NA | 0.45  (-1.65, 2.52) | NA |
|  | High-dose carbohydrate vs Carbohydrate, iv | NA | NA | -1.02  (-3.15, 1.13) | NA |
|  | High-dose carbohydrate vs Placebo | 0.03  (-0.56,0.65) | NA | 0.02  (-0.56, 0.66) | NA |
|  | High-dose carbohydrate vs Fasting | 0.09  (-1.10,1.30) | NA | 0.16  (-0.91, 1.28) | NA |
| Postoperative FPG | Low-dose carbohydrate vs Carbohydrate, iv | 0.10  (-1.11,1.30) | -0.70  (-1.80,0.37) | -0.82  (-1.55, -0.11) | 0.08 |
|  | Low-dose carbohydrate vs Placebo | 0.62  (-0.04, 1.30) | 0.25  (-0.50, 0.99) | -0.44  (-0.92, 0.04) | 0.45 |
|  | Low-dose carbohydrate vs Fasting | 0.43  (-0.01, 0.88) | 1.90  (0.70, 3.20) | -0.56  (-0.99, -0.14) | 0.03* |
|  | High-dose carbohydrate vs Carbohydrate, iv | 0.10  (-1.10, 1.30) | 0.67  (-0.23, 1.60) | -0.40  (-1.08, 0.26) | 0.44 |
|  | High-dose carbohydrate vs Placebo | 0.05  (-0.40, 0.50) | -0.02  (-0.78, 0.74) | -0.03  (-0.38, 0.33) | 0.88 |
|  | High-dose carbohydrate vs Fasting | 0.17  (-0.19, 0.55) | 0.08  (-0.77, 0.94) | -0.15  (-0.46, 0.16) | 0.83 |
|  | Carbohydrate, iv vs Placebo | -2.00  (-3.20, -0.82) | 0.21  (-0.48, 0.92) | 0.38  (-0.30, 1.07) | 0.01* |
|  | Carbohydrate, iv vs Fasting | 0.17  (-0.56, 0.92) | -2.00  (-3.20, -0.74) | 0.26  (-0.39, 0.92) | 0.01* |
| Postoperative Fins | Low-dose carbohydrate vs Placebo | -5.34  (-18.30,0.79) | -5.91  (-15.60,3.80) | -5.48  (-10.83, -0.18) | 0.92 |
|  | Low-dose carbohydrate vs Fasting | -3.48  (-8.71,1.76) | -13.20  (-26.10,0.06) | -4.42  (-9.21, 0.37) | 0.16 |
|  | High-dose carbohydrate vs Placebo | -2.38  (-7.20,2.33) | -2.93  (-11.40,5.69) | -2.46  (-6.42, 1.4) | 0.91 |
|  | High-dose carbohydrate vs Fasting | -1.32  (-5.44,2.82) | -1.54  (-10.50,7.36) | -1.42  (-4.95, 2.10) | 0.96 |
| the serum levels of CRP | Low-dose carbohydrate vs Placebo | 24.3  (-25.50,73.90) | -17.50  (-75.10,41.70) | 5.83  (-31.11, 45.84) | 0.26 |
|  | Low-dose carbohydrate vs Fasting | -27.20  (-70.20,16.50) | 14.4  (-48.90,75.60) | -14.25  (-50.60, 22.64) | 0.26 |
|  | High-dose carbohydrate vs Placebo | -7.10  (-36.10,22.6) | 34.4  (-36.00,103.00) | -1.42  (-28.28, 27.30) | 0.26 |
|  | High-dose carbohydrate vs Fasting | -16.90  -42.90,7.25) | -58.60  (-130.00,13.40) | -21.28  (-46.71, 1.84) | 0.25 |
| Postoperative pain | Low-dose carbohydrate vs Placebo | -0.51  (-6.73,5.91) | -1.37  (-10.80,7.96) | -0.77  (-5.53, 4.02) | 0.85 |
|  | Low-dose carbohydrate vs Fasting | -1.81  (-8.18,4.53) | -0.92  (-10.30,8.39) | -1.52  (-6.32, 3.25) | 0.85 |
|  | High-dose carbohydrate vs Placebo | -0.71  (-6.98,5.63) | 0.15  (-9.22,9.57) | -0.41  (-5.20, 4.34) | 0.86 |
|  | High-dose carbohydrate vs Fasting | -1.13  (-3.96,1.71) | -2.00  (-13.00,8.96) | -1.16  (-3.68, 1.29) | 0.85 |
|  | Carbohydrate, iv vs Placebo | NA | NA | 0.50  (-6.08, 7.13) | NA |
|  | †Carbohydrate, iv vs Fasting | -0.40  (-2.50,1.70) | NA | -0.25  (-5.84, 5.34) | NA |
| Postoperative patient satisfaction | Low-dose carbohydrate vs Placebo | NA | NA | 5.25  (-2.00, 12.5) | NA |
|  | Low-dose carbohydrate vs Fasting | NA | NA | 3.26  (-1.95, 8.46) | NA |
|  | High-dose carbohydrate vs Placebo | NA | NA | 4.00  (-1.02, 9.01) | NA |
|  | High-dose carbohydrate vs Fasting | NA | NA | 2.00  (-2.99, 7.04) | NA |
| Postoperative thirst | Low-dose carbohydrate vs Placebo | -0.88  (-9.23,7.23) | NA | -0.90  (-9.14, 7.43) | NA |
|  | Low-dose carbohydrate vs Fasting | NA | NA | -3.35  (-14.46, 7.68) | NA |
|  | High-dose carbohydrate vs Placebo | 1.00  (-7.31,9.35) | NA | 0.59  (-6.78, 8.04) | NA |
|  | High-dose carbohydrate vs Fasting | -1.88  (-5.62,1.87) | NA | -1.87  (-5.61, 1.85) | NA |
| Postoperative hungry | Low-dose carbohydrate vs Placebo | -0.63  (-8.44,7.01) | NA | -0.69  (-8.46, 7.07) | NA |
|  | Low-dose carbohydrate vs Fasting | NA | NA | -2.24  (-12.64, 8.2) | NA |
|  | High-dose carbohydrate vs Placebo | 0.51  (-7.20,8.24) | NA | 0.43  (-6.52, 7.35) | NA |
|  | High-dose carbohydrate vs Fasting | -1.13  (-4.61,2.33) | NA | -1.13  (-4.64, 2.34) | NA |
| Postoperative anxiety | Low-dose carbohydrate vs Placebo | 0.10  (-8.66,8.80) | NA | 0.09  (-8.59, 8.80) | NA |
|  | Low-dose carbohydrate vs Fasting | NA | NA | -2.52  (-14.48, 9.46) | NA |
|  | High-dose carbohydrate vs Placebo | 1.49  (-7.20,10.20) | NA | -0.11  (-8.25, 8.02) | NA |
|  | High-dose carbohydrate vs Fasting | 0.50  (-0.27,1.27) | NA | -2.72  (-8.88, 3.45) | NA |
| Postoperative nausea and vomit | Low-dose carbohydrate vs Carbohydrate, iv | -0.27  (-2.02,1.49) | NA | -0.26  (-2.04,1.51) | NA |
|  | Low-dose carbohydrate vs Placebo | NA | NA | -1.78  (-4.12, 0.53) | NA |
|  | Low-dose carbohydrate vs Fasting | -1.36  (-3.45,0.72) | NA | -1.36  (-3.43, 0.72) | NA |
|  | High-dose carbohydrate vs Carbohydrate, iv | NA | NA | 0.75  (-1.54,3.04) | NA |
|  | High-dose carbohydrate vs Placebo | -0.85  (-1.92,0.10) | NA | -0.76  (-1.76, 0.16) | NA |
|  | †High-dose carbohydrate vs Fasting | -0.10  (-0.34,0.14) | NA | -0.35  (-1.16, 0.46) | NA |
|  | Carbohydrate, iv vs Placebo | NA | NA | -1.52  (-3.92,0.84) | NA |
|  | Carbohydrate, iv vs Fasting | -1.15  (-9.54,7.44) | NA | -1.10  (-3.24,1.05) | NA |
| Occurrences of postoperative infection | Low-dose carbohydrate vs Placebo | NA | NA | -0.54  (-1.78, 0.66) | NA |
|  | Low-dose carbohydrate vs Fasting | -2.40  (-5.30,1.10) | NA | 0.42  (0.20,0.81) | NA |
|  | High-dose carbohydrate vs Placebo | -1.10  (-2.30,0.52) | NA | 0.93  (0.42,1.70) | NA |
|  | High-dose carbohydrate vs Fasting | -1.60  (-4.30,0.64) | NA | 0.71  (0.37,1.30) | NA |

NA: not applicable.

* Incoherence between direct and indirect estimate.

†The indirect widens the credible interval from the network in comparison to the direct more than it should.

Treatment estimates are odds ratios(ORs) and 95% confidence intervals(Crls) for occurrences of postoperative infection, and mean difference(MDs) and 95% confidence intervals(Crls) for the remaining outcomes.

**1.8 Supplementary Table S8.** Cumulative ranking of interventions for secondary outcomes

| **Outcome** | **Rank** | **Intervention** | **SUCRA** |
| --- | --- | --- | --- |
| RGV | 1 | Low-dose carbohydrate | 53.1% |
|  | 2 | High-dose carbohydrate | 31.5% |
|  | 3 | Placebo | 9.5% |
|  | 4 | Fasting | 5.9% |
| Postoperative insulin sensitivity | 1 | Fasting | 42.2% |
|  | 2 | Low-dose carbohydrate | 25.4% |
|  | 3 | Placebo | 13.3% |
|  | 4 | High-dose carbohydrate | 12.2% |
|  | 5 | Carbohydrate, iv | 6.76% |
| Postoperative FPG | 1 | Low-dose carbohydrate | 92.7% |
|  | 2 | High-dose carbohydrate | 3.6% |
|  | 3 | Placebo | 2.4% |
|  | 4 | Carbohydrate, iv | 1.0% |
|  | 5 | Fasting | 0.1% |
| Postoperative Fins | 1 | Low-dose carbohydrate | 85.8% |
|  | 2 | High-dose carbohydrate | 12.2% |
|  | 3 | Fasting | 1.2% |
|  | 4 | Placebo | 0.7% |
|  | 5 | Carbohydrate, iv | 2.9% |
| the serum levels of CRP | 1 | Fasting | 73.1% |
|  | 2 | Low-dose carbohydrate | 18.3% |
|  | 3 | Placebo | 7.0% |
|  | 4 | High-dose carbohydrate | 1.6% |
| Postoperative pain | 1 | Low-dose carbohydrate | 35.8% |
|  | 2 | High-dose carbohydrate | 25.7% |
|  | 3 | Placebo | 18.4% |
|  | 4 | Carbohydrate, iv | 17.3% |
|  | 5 | Fasting | 2.8% |
| Postoperative patient satisfaction | 1 | Placebo | 81.9% |
|  | 2 | Fasting | 12.0% |
|  | 3 | Low-dose carbohydrate | 3.3% |
|  | 4 | High-dose carbohydrate | 2.7% |
| Postoperative thirst | 1 | Low-dose carbohydrate | 50.0% |
|  | 2 | High-dose carbohydrate | 24.5% |
|  | 3 | Placebo | 22.2% |
|  | 4 | Fasting | 3.3% |
| Postoperative hungry | 1 | Low-dose carbohydrate | 47.2% |
|  | 2 | High-dose carbohydrate | 24.3% |
|  | 3 | Placebo | 21.9% |
|  | 4 | Fasting | 6.6% |
| Postoperative anxiety | 1 | Low-dose carbohydrate | 36.1% |
|  | 2 | High-dose carbohydrate | 34.7% |
|  | 3 | Placebo | 23.6% |
|  | 4 | Fasting | 5.6% |
| Postoperative nausea and vomit | 1 | Low-dose carbohydrate | 56.1% |
|  | 2 | Carbohydrate, iv | 36.1% |
|  | 3 | High-dose carbohydrate | 10.6% |
|  | 4 | Fasting | 1.0% |
|  | 5 | Placebo | 0.6% |
| Postoperative fatigue | 1 | Low-dose carbohydrate | 58.1% |
|  | 2 | High-dose carbohydrate | 22.3% |
|  | 3 | Placebo | 12.2% |
|  | 4 | Fasting | 7.4% |
| Postoperative weakness | 1 | Placebo | 66.7% |
|  | 2 | Fasting | 28.1% |
|  | 3 | High-dose carbohydrate | 5.1% |
| Postoperative infection | 1 | Low-dose carbohydrate | 75.5% |
|  | 2 | High-dose carbohydrate | 14.3% |
|  | 3 | Placebo | 10.1% |
|  | 4 | Fasting | 0.2% |

SUCRA: surface under the cumulative ranking curve;

RGV: residual gastric volume; FPG: fasting plasma glucose; Fins: fasting serum insulin; CRP: C-reactive protein.

**1.9 Supplementary Table S9.** Network meta-regression for different covariates of secondary outcomes

| **Outcome** |  |  | **50% (95%Crl)** |
| --- | --- | --- | --- |
| RGV | Adjusted for the risk of bias | Beta[1] | -0.38 (-37.29,26.31) |
|  |  | Beta[2] | -0.13 (-40.24,25.68) |
|  |  | Beta[3] | 0.31 (-23.69,50.01) |
|  |  | Beta[4] | -1.69 (-55.03,2.07) |
|  | Adjusted for blinding | Beta[1] | -0.26 (-23.95,20.72) |
|  |  | Beta[2] | -0.13 (-21.84,20.75) |
|  |  | Beta[3] | -3.30 (-15.29,4.02) |
|  |  | Beta[4] | -2.32 (-17.64,10.88) |
|  | Adjusted for the type of surgery | Beta[1] | -0.25 (-23.82,20.94) |
|  |  | Beta[2] | -0.13 (-25.71,20.36) |
|  |  | Beta[3] | -1.45 (-53.66,17.73) |
|  |  | Beta[4] | 0.68 (-22.89,34.11) |
| Postoperative insulin sensitivity | Adjusted for the risk of bias | Beta[1] | -0.45 (-1.52,0.56) |
|  |  | Beta[2] | -0.27 (-2.47,2.02) |
|  |  | Beta[3] | -0.002 (-13.47,13.13) |
|  |  | Beta[4] | 0.01 (-14.24,17.89) |
|  |  | Beta[5] | -0.11 (-6.09,6.90) |
|  |  | Beta[6] | 0.25 (-1.90,2.68) |
|  | Adjusted for blinding | Beta[1] | -0.18 (-1.44,1.12) |
|  |  | Beta[2] | -0.13 (-2.64,2.02) |
|  |  | Beta[3] | 0.03 (-13.27,52.66) |
|  |  | Beta[4] | -0.01 (-9.90,8.90) |
|  |  | Beta[5] | 0.12 (-3.80,13.38) |
|  |  | Beta[6] | 0.05 (-2.30,2.28) |
|  | Adjusted for the type of surgery | Beta[1] | 0.15 (-1.15,1.44) |
|  |  | Beta[2] | 0.09 (-2.17,2.64) |
|  |  | Beta[3] | 0.02 (-17.34,11.32) |
|  |  | Beta[4] | -0.07 (-16.50,8.07) |
|  |  | Beta[5] | 0.06 (-8.60,8.45) |
|  |  | Beta[6] | -0.07 (-2.43,2.37) |
| Postoperative FPG | Adjusted for the risk of bias | Beta[1] | -0.54 (-1.54,0.48) |
|  |  | Beta[2] | 0.13 (-0.67,0.94) |
|  |  | Beta[3] | -0.36 (-1.07,0.31) |
|  |  | Beta[4] | 0.26 (-0.33,0.85) |
|  |  | Beta[5] | -0.61(-1.35,0.12) |
|  |  | Beta[6] | 0.63 (-0.12,1.40) |
|  | Adjusted for blinding | Beta[1] | 0.36 (-0.50,1.26) |
|  |  | Beta[2] | 0.35 (-0.41,1.14) |
|  |  | Beta[3] | 0.11 (-0.59,0.74) |
|  |  | Beta[4] | 0.10 (-0.55,0.81) |
|  |  | Beta[5] | 0.04 (-0.70,0.74) |
|  |  | Beta[6] | 0.01 (-0.69,0.81) |
|  | Adjusted for the type of surgery | Beta[1] | 0.17 (-0.46,0.75) |
|  |  | Beta[2] | 0.22 (-0.40,0.82) |
|  |  | Beta[3] | -0.34 (-1.20,0.47) |
|  |  | Beta[4] | -0.29 (-1.04,0.40) |
|  |  | Beta[5] | -0.09 (-0.73,0.54) |
|  |  | Beta[6] | 0.03 (-0.56,0.66) |
| Postoperative FIns | Adjusted for the risk of bias | Beta[1] | -5.95 (-22.23,4.37) |
|  |  | Beta[2] | -5.52 (-22.13,5.80) |
|  |  | Beta[3] | 0.19 (-39.42,7.85) |
|  |  | Beta[4] | 1.42 (-29.08,7.40) |
|  |  | Beta[5] | 1.09 (-133.04,538.82) |
|  |  | Beta[6] | 0.38 (-182.47,163.13) |
|  | Adjusted for blinding | Beta[1] | 2.09 (-26.07,15.80) |
|  |  | Beta[2] | -2.58 (-13.73,15.20) |
|  |  | Beta[3] | 3.41 (-9.60,12.21) |
|  |  | Beta[4] | 1.05 (-10.03,18.41) |
|  |  | Beta[5] | 5.72 (-94.19,236.18) |
|  |  | Beta[6] | -2.71 (-298.16,84.96) |
|  | Adjusted for the type of surgery | Beta[1] | 7.60 (-5.27,20.67) |
|  |  | Beta[2] | 3.32 (-8.44,19.99) |
|  |  | Beta[3] | 2.24 (-4.23,8.63) |
|  |  | Beta[4] | 0.32 (-36.90,63.78) |
|  |  | Beta[5] | -0.27 (-105.81,114.05) |
|  |  | Beta[6] | -1.20 (-99.80,67.67) |
| CRP | Adjusted for the risk of bias | Beta[1] | 15.39 (-48.87,110.58) |
|  |  | Beta[2] | 23.53 (-46.94,132.21) |
|  |  | Beta[3] | -2.85 (-40.62,36.51) |
|  |  | Beta[4] | 7.02 (-79.31,40.68) |
|  | Adjusted for blinding | Beta[1] | -15.91 (-79.09,32.96) |
|  |  | Beta[2] | -12.75 (-83.31,41.03) |
|  |  | Beta[3] | 12.70 (-21.68,39.77) |
|  |  | Beta[4] | 15.69 (-23.76,70.51) |
|  | Adjusted for the type of surgery | Beta[1] | -16.71 (-92.30,42.62) |
|  |  | Beta[2] | -20.92 (-97.28,30.19) |
|  |  | Beta[3] | 5.23 (-34.28,94.06) |
|  |  | Beta[4] | -4.87 (-52.73,33.82) |
| Postoperative pain | Adjusted for the risk of bias | Beta[1] | -0.45 (-6.26,6.64) |
|  |  | Beta[2] | -0.51 (-5.66,5.61) |
|  |  | Beta[3] | 2.86 (-6.09,8.89) |
|  |  | Beta[4] | 3.28 (-0.22,6.74) |
|  |  | Beta[5] | -0.26 (-31.51,20.40) |
|  |  | Beta[6] | -0.07 (-57,05,73.16) |
|  | Adjusted for blinding | Beta[1] | 0.29 (-15.20,6.13) |
|  |  | Beta[2] | 1.42 (-3.19,28.39) |
|  |  | Beta[3] | -1.49 (-21.41,6.96) |
|  |  | Beta[4] | 0.98 (-5.42,18.20) |
|  |  | Beta[5] | 0.16 (-26.64,65.86) |
|  |  | Beta[6] | 0.18 (-28.34,37,46) |
|  | Adjusted for the type of surgery | Beta[1] | -0.43 (-13.96,11.91) |
|  |  | Beta[2] | -1.94 (-14.50,12.60) |
|  |  | Beta[3] | 0.35 (-5.46,5.17) |
|  |  | Beta[4] | 1.02 (-2.94,4.59) |
|  |  | Beta[5] | 0.14 (-38.57,97.97) |
|  |  | Beta[6] | -0.10 (-44.11,45.03) |
| Postoperative patient satisfaction | Adjusted for the risk of bias | Beta[1] | -0.40 (-18.57,21.00) |
|  |  | Beta[2] | -0.40 (-18.57,21.00) |
|  |  | Beta[3] | -0.65 (-8.37,6.76) |
|  |  | Beta[4] | -0.65 (-8.37,6.76) |
|  | Adjusted for blinding | All are open labels | |
|  | Adjusted for the type of surgery | Beta[1] | -0.013 (-21.79,45.53) |
|  |  | Beta[2] | -1.31 (-54.12,17.27) |
|  |  | Beta[3] | 0.80 (-8.41,10.39) |
|  |  | Beta[4] | 2.16 (-9.74,23.88) |
| Postoperative thirst | Adjusted for the risk of bias | Beta[1] | -0.52 (-48.13,27.15) |
|  |  | Beta[2] | -19.42 (-68.82,22.82) |
|  |  | Beta[3] | 1.27 (-9.10,11.17) |
|  |  | Beta[4] | 3.10 (-2.98,8.99) |
|  | Adjusted for blinding | Beta[1] | 9.70 (-8.71,53.94) |
|  |  | Beta[2] | 13.77 (-5.49,30.55) |
|  |  | Beta[3] | -0.27 (-79.45,52.39) |
|  |  | Beta[4] | 0.24 (-38.31,56.89) |
|  | Adjusted for the type of surgery | Beta[1] | -15.09 (-41.64,6.92) |
|  |  | Beta[2] | -1.24 (-65.16,51.31) |
|  |  | Beta[3] | 0.58 (-10.71,12.60) |
|  |  | Beta[4] | 1.09 (-6.85,8.91) |
| Postoperative hungry | Adjusted for the risk of bias | Beta[1] | 0.19 (-19.07,18.17) |
|  |  | Beta[2] | 2.19 (-15.00,25.17) |
|  |  | Beta[3] | 2.07 (-8.93,12.64) |
|  |  | Beta[4] | 3.84 (-1.40,7.97) |
|  | Adjusted for blinding | Beta[1] | -2.98 (-9.57,5.34) |
|  |  | Beta[2] | -2.74 (-38.32,7.63) |
|  |  | Beta[3] | 0.29 (-23.87,31.99) |
|  |  | Beta[4] | 0.18 (-18.61,21.81) |
|  | Adjusted for the type of surgery | Beta[1] | -2.68 (-13.34,33.59) |
|  |  | Beta[2] | -5.57 (-11.50,13.13) |
|  |  | Beta[3] | 1.12 (-11.86,17.09) |
|  |  | Beta[4] | 1.79 (-4.91,8.50) |
| Postoperative anxiety | Adjusted for the risk of bias | Beta[1] | 6.25 (-13.64,27.29) |
|  |  | Beta[2] | 1.41 (-13.49,15.42) |
|  |  | Beta[3] | 2.24 (-9.26,13.14) |
|  |  | Beta[4] | 4.96 (-4.41,12.96) |
|  | Adjusted for blinding | Beta[1] | 1.74 (-20.05,17.07) |
|  |  | Beta[2] | 12.90 (7.46,32.62) |
|  |  | Beta[3] | -0.52 (-30.85,24.31) |
|  |  | Beta[4] | -0.11 (-41.25,28.46) |
|  | Adjusted for the type of surgery | Beta[1] | 5.50 (-27.07,24.81) |
|  |  | Beta[2] | -1.46 (-24.89,22.91) |
|  |  | Beta[3] | 0.10 (-27.72,26.98) |
|  |  | Beta[4] | 0.10 (-31.69,51.68) |
| Postoperative nausea and vomit | Adjusted for the risk of bias | Beta[1] | -0.10 (-21.67,13.80) |
|  |  | Beta[2] | 0.13 (-19.06,18.68) |
|  |  | Beta[3] | 0.28 (-1.18,1.98) |
|  |  | Beta[4] | 0.18 (-1.78,2.30) |
|  |  | Beta[5] | 0.05 (-22.19,17.33) |
|  |  | Beta[6] | -0.04 (-17.52,15.40) |
|  | Adjusted for blinding | Beta[1] | 0.07 (-13.99,23.56) |
|  |  | Beta[2] | -0.003 (-14.32,15.67) |
|  |  | Beta[3] | -0.16 (-1.57,1.47) |
|  |  | Beta[4] | 0.06 (-16.42,19.05) |
|  |  | Beta[5] | -0.04 (-22.24,16.91) |
|  |  | Beta[6] | 0.14 (-2.85,2.44) |
|  | Adjusted for the type of surgery | Beta[1] | -0.01 (-22.58,21.81) |
|  |  | Beta[2] | 0.07 (-16.28,20.33) |
|  |  | Beta[3] | 0.07 (-2.29,2.19) |
|  |  | Beta[4] | 0.31(-1.39,1.87) |
|  |  | Beta[5] | 0.07 (-13.58,17.03) |
|  |  | Beta[6] | -0.03 (-45.32,23.99) |
| Postoperative fatigue | Adjusted for the risk of bias | Beta[1] | -0.34 (-11.20,5.48) |
|  |  | Beta[2] | -0.58 (-22.19,36.06) |
|  |  | Beta[3] | 0.77 (-3.80,5.90) |
|  |  | Beta[4] | 1.40 (-1.74,4.50) |
|  | Adjusted for blinding | Beta[1] | 0.21 (-7.57,8.03) |
|  |  | Beta[2] | -0.08 (-6.63,5.43) |
|  |  | Beta[3] | -0.09 (-23.80,12.95) |
|  |  | Beta[4] | -0.10 (-10.66,10.21) |
|  | Adjusted for the type of surgery | Beta[1] | -0.20 (-5.14,6.65) |
|  |  | Beta[2] | 1.04 (-3.23,17.04) |
|  |  | Beta[3] | 0.94 (-4.30,7.45) |
|  |  | Beta[4] | -0.18 (-7.59,6.02) |
| Postoperative weakness | Adjusted for the risk of bias | Beta[3] | -0.43 (-3.15,1.83) |
|  |  | Beta[4] | -0.77 (-2.76,0.99) |
|  | Adjusted for blinding | All are open labels |  |
|  | Adjusted for type of surgery | Beta[3] | -0.44 (-3.18,1,80) |
|  |  | Beta[4] | -0.76 (-2.71,1.01) |
| Occurrences of postoperative infection | Adjusted for the risk of bias | Beta[1] | 0.48 (-1.35,2.67) |
|  |  | Beta[2] | 0.77 (-0.97,2.85) |
|  |  | Beta[3] | 0.04 (-0.88,1.05) |
|  |  | Beta[4] | 0.35 (-0.75,1.51) |
|  | Adjusted for blinding | Beta[1] | -0.76 (-3.17,1.22) |
|  |  | Beta[2] | -0.51 (-2.91,1.06) |
|  |  | Beta[3] | 0.15 (-1.00,1.56) |
|  |  | Beta[4] | 0.49 (-1.00,2.27) |
|  | Adjusted for the type of surgery | Beta[1] | 0.09 (-1.91,2.02) |
|  |  | Beta[2] | 0.49 (-0.83,1.89) |
|  |  | Beta[3] | 0.14 (-1.33,1.83) |
|  |  | Beta[4] | 0.44 (-1.30,2.52) |

Crls= Credible intervals; RGV: residual gastric volume; FPG: fasting plasma glucose; Fins: fasting serum insulin; CRP: C-reactive protein.

[1] Low-dose carbohydrate versus Placebo/Water ; [2] Low-dose carbohydrate versus Fasting; [3] High-dose carbohydrate versus Placebo/Water;

[4] High-dose carbohydrate versus Fasting; [5] Carbohydrate, iv versus Placebo/water; [6] Carbohydrate, iv versus Fasting;

**1.10 Supplementary Table S10.**

summary of results for the sensitivity analysis using a Fixed effect model instead of the Random effect model used as main analysis

| **Outcomes** | **Treatments** | **Main analysis**  **(Random effect model)** | | | **Sensitivity analysis**  **(Fixed effect model)** | |
| --- | --- | --- | --- | --- | --- | --- |
|  |  | **vs Placebo/Water** | **vs Fasting** | | **vs Placebo/Water** | **vs Fasting** |
| HOMA-IR¶ | Low-dose carbohydrate | -3.02 (-4.80, -1.26) | -1.72 (-3.33, -0.18) | | 0.04 (-0.02, 0.09) | -0.12 (-0.18, -0.06) |
|  | High-dose carbohydrate | -1.90 (-3.43, -0.33) | -0.60 (-1.74, 0.54) | | -0.94 (-1.07, -0.8) | -1.1 (-1.22, -0.97) |
|  |  | DIC: 101.15 | | | DIC: 1100.78 | |
| RGV¶ | Low-dose carbohydrate | -2.81 (-11.93, 7.54) | -2.41 (-9.99, 5.31) | | -3.37 (-7.89, 1.15) | -2.37 (-6.86, 2.12) |
|  | High-dose carbohydrate | -2.02 (-6.35, 4.84) | -1.61 (-7.2, 5.44) | | -2.98 (-3.38, -2.59) | -1.99 (-2.56, -1.42) |
|  |  | DIC: 16.18 | | | DIC: 17.17 | |
| Postoperative insulin sensitivity¶ | Low-dose carbohydrate | 0.30 (-1.49, 2.09) | | 1.05 (-1.08, 3.20) | 0.30 (-1.08, 1.68) | 0.49 (-0.90, 1.88) |
|  | High-dose carbohydrate | 0.02 (-0.56, 0.66) | | 0.16 (-0.91, 1.28) | -0.07 (-0.23, 0.09) | 0.12 (-0.05, 0.30) |
|  | Carbohydrate, iv | 1.05 (-1.08, 3.20) | | 1.20(-0.66, 3.05) | 1.00 (-0.47, 2.48) | 0.19 (0.01, 0.37) |
|  |  | DIC: 29.58 | | | DIC: 31.53 | |
| Postoperative FPG¶ | Low-dose carbohydrate | -0.82 (-1.55, -0.11) | -0.56 (-0.99, -0.14) | | -0.41 (-0.55, -0.27) | -0.4 (-0.54, -0.26) |
|  | High-dose carbohydrate | -0.03 (-0.38, 0.33) | -0.15 (-0.46, 0.16) | | 0 (-0.07, 0.06) | 0.01 (-0.07, 0.09) |
|  | Carbohydrate, iv | 0.38 (-0.30, 1.07) | 0.26 (-0.39, 0.92) | | 0.2 (-0.11, 0.51) | 0.21 (-0.1, 0.52) |
|  |  | DIC: 117.10 | | | DIC: 226.73 | |
| Postoperative Fins¶ | Low-dose carbohydrate | -5.48 (-10.83, -0.18) | -4.42 (-9.21, 0.37) | | -0.16 (-0.43, 0.11) | -1.24 (-1.54, -0.94) |
|  | High-dose carbohydrate | -2.46 (-6.42, 1.40) | -1.42 (-4.95, 2.10) | | 0.13 (-0.21, 0.47) | -0.96 (-1.24, -0.68) |
|  | Carbohydrate, iv | 12.50 (0.9, 24.10) | 13.56 (1.22, 25.97) | | 12.5 (10.96, 14.04) | 11.41 (9.86, 12.97) |
|  |  | DIC: 93.24 | | | DIC: 782.98 | |
| CRP¶ | Low-dose carbohydrate | 5.83 (-31.11, 45.84) | -14.25 (-50.6, 22.64) | | -18.39 (-31.31, 30.69) | -10.45 (-23.44, 35.98) |
|  | High-dose carbohydrate | -1.42 (-28.28, 27.3) | -21.28 (-46.71, 1.84) | | -8.51 (-15.51, 3.03) | -0.86 (-1.16, -0.57) |
|  |  | DIC: 26.81 | | | DIC: 96.31 | |
| Postoperative pain¶ | Low-dose carbohydrate | 0.30 (-1.49, 2.09) | 0.45 (-1.65, 2.52) | | -0.51 (-0.55, -0.46) | 0.73 (0.58, 0.88) |
|  | High-dose carbohydrate | 0.02 (-0.56, 0.66) | 0.16 (-0.91, 1.28) | | -0.67 (-0.76, -0.57) | 0.57 (0.46, 0.68) |
|  | Carbohydrate, iv | 1.05 (-1.08, 3.20) | 1.20 (-0.66, 3.05) | | 0.43 (-0.48, 1.33) | 1.67 (0.75, 2.58) |
|  |  | DIC: 34.03 | | | DIC: 278.72 | |
| Postoperative patient satisfaction¶ | Low-dose carbohydrate | 5.25 (-2.00, 12.50) | 3.26 (-1.95, 8.46) | | 5.23 (3.65, 6.85) | 3.23 (1.65, 4.85) |
|  | High-dose carbohydrate | 4.00 (-1.02, 9.01) | 2.00 (-2.99, 7.04) | | 4.00 (3.72, 4.27) | 2.00 (1.68, 2.31) |
|  |  | DIC:10.01 | | | DIC:11.98 | |
| Postoperative thirst¶ | Low-dose carbohydrate | -0.90 (-9.14, 7.43) | -3.35 (-14.46, 7.68) | | -0.90 (-0.98, -0.82) | -3.12 (-3.68, -2.55) |
|  | High-dose carbohydrate | 0.59 (-6.78, 8.04) | -1.87 (-5.61, 1.85) | | 0.90 (0.37, 1.44) | -1.31 (-1.57, -1.05) |
|  |  | DIC:26.01 | | | DIC: 152.87 | |
| Postoperative hungry¶ | Low-dose carbohydrate | -0.69 (-8.46, 7.07) | -2.24 (-12.64, 8.2) | | -0.70 (-0.78, -0.62) | -1.04 (-1.63, -0.43) |
|  | High-dose carbohydrate | 0.43 (-6.52, 7.35) | -1.13 (-4.64, 2.34) | | 0.89 (0.31, 1.46) | 0.55 (0.36, 0.75) |
|  |  | DIC:26.05 | | | DIC:180.20 | |
| Postoperative anxiety¶ | Low-dose carbohydrate | 0.09 (-8.59, 8.8) | -2.52 (-14.48, 9.46) | | 0.10 (0.04, 0.16) | -2.14 (-2.69, -1.58) |
|  | High-dose carbohydrate | -0.11 (-8.25, 8.02) | -2.72 (-8.88, 3.45) | | 1.08 (0.66, 1.50) | -1.15 (-1.67, -0.64) |
|  |  | DIC:14.01 | | | DIC: 130.50 | |
| Postoperative nausea and vomit¶ | Low-dose carbohydrate | -1.78 (-4.12, 0.53) | -1.36 (-3.43, 0.72) | | -1.58 (-2.87, -0.31) | -1.36 (-2.63, -0.1) |
|  | High-dose carbohydrate | -0.76 (-1.76, 0.16) | -0.35 (-1.16, 0.46) | | -0.44 (-0.6, -0.28) | -0.22 (-0.38, -0.06) |
|  |  | DIC:32.96 | | | DIC:58.46 | |
| Postoperative fatigue¶ | Low-dose carbohydrate | -0.70 (-3.65, 2.26) | -1.49 (-6.53, 3.12) | | -0.70 (-0.78, -0.62) | -1.05 (-2.17, 0.07) |
|  | High-dose carbohydrate | 0.00 (-3.06, 3.08) | -0.81 (-3.23, 1.25) | | 0.00 (-0.9, 0.9) | -0.35 (-1.02, 0.31) |
|  |  | DIC:16.47 | | | DIC: 19.65 | |
| Postoperative weakness¶ | Low-dose carbohydrate | - | - | | - | - |
|  | High-dose carbohydrate | 0.68 (-0.69, 2.12) | 0.37 (-0.56, 1.47) | | 0.53 (-0.10, 1.17) | 0.09 (-0.13, 0.31) |
|  |  | DIC:10.42 | | | DIC:14.56 | |
| Occurrences of postoperative infection**#** | Low-dose carbohydrate | -0.54 (-1.78, 0.66) | 0.42 (0.20,0.81) | | 0.58 (0.22,1.50) | 0.42 (0,.20,0.80) |
|  | High-dose carbohydrate | 0.93 (0.42,1.70) | 0.71 (0.37,1.30) | | 0.98 (0.68,1.40) | 0.70 (0.37,1.30) |
|  |  | DIC: 35.63 | | | DIC: 33.69 | |

Data are mean differences (MDs) and 95% credibility intervals (Crsl);

HOMA-IR: homeostasis model assessment-insulin resistance; FPG: fasting plasma glucose; Fins: fasting insulin level; CRP: C-reactive protein.

#: Odds ratios(ORs)；

-: No data available for this outcome.

**1.11 Supplementary Table S11.** summary of results for the sensitivity analyses

| Outcome | Treatment | Main analyses | | sensitivity analyses | | | No high risk of bias trials and imputed data | |
| --- | --- | --- | --- | --- | --- | --- | --- | --- |
|  |  | Placebo/Water | Fasting | Placebo | Water | Fasting | Placebo/Water | Fasting |
| HOMA-IR¶ | Low-dose carbohydrate | -3.02  (-4.80,-1.26) | -1.72  (-3.33,-0.18) | -3.98  (-6.04, -1.97) | -1.64  (-3.97, 0.65) | -1.45  (-3.02, 0.07) | -1.24  (-2.32,-0.16) | -1.20  (-1.99,-0.41) |
|  | High-dose carbohydrate | -1.90  (-3.43,-0.33) | -0.60  (-1.74,0.54) | -3.27  (-5.44, -1.10) | -0.93  (-2.78, 0.92) | -0.74  (-1.84, 0.37) | -0.51  (-1.59,0.59) | -0.47  (-1.17,0.59) |
| RGV¶ | Low-dose carbohydrate | -2.81  (-11.93, 7.54) | -2.41  (-9.99, 5.31) | -3.26  (-12.64, 6.41) | 4.68  (-9.63,19.29) | -2.29  (-9.64, 5.13) | 1.51  (-48.88, 52.92) | - |
|  | High-dose carbohydrate | -2.02  (-6.35, 4.84) | -1.61  (-7.20, 5.44) | -2.98  (-9.23, 3.34) | 4.98  (-5.96, 15.93) | -2.01  (-8.36, 4.19) | 5.03  (-5.89, 16.03) | - |
| Postoperative insulin sensitivity¶ | Low-dose carbohydrate | 0.30  (-1.49, 2.09) | 0.45  (-1.65, 2.52) | 0.25  (-0.53, 1.15) | -0.26  (-1.4, 0.71) | 0.04  (-1.27, 1.25) | 0.25  (-0.44, 1.03) | - |
|  | High-dose carbohydrate | 0.02  (-0.56, 0.66) | 0.16  (-0.91, 1.28) | 0.29  (-1.57, 2.15) | -0.24  (-2.63, 1.94) | 0.06  (-2.4, 2.36) | - | - |
|  | Carbohydrate, iv | 1.05  (-1.08, 3.20) | 1.20  (-0.66, 3.05) | 1.42  (-0.97, 3.93) | 0.89  (-1.45, 3.15) | 1.19  (-0.76, 3.12) | - | - |
| Postoperative FPG**¶** | Low-dose carbohydrate | -0.44  (-0.92, 0.04) | **-0.56**  **(-0.99, -0.14)** | -0.60  (-1.21, 0.00) | -0.31  (-0.88, 0.29) | **-0.54**  **(-0.98, -0.11)** | -0.22  (-0.60, 0.23) | **-0.59**  **(-0.98, -0.24)** |
|  | High-dose carbohydrate | -0.03  (-0.38, 0.33) | -0.15  (-0.46, 0.16) | -0.21 (-0.77, 0.33) | 0.08  (-0.35, 0.54) | -0.15  (-0.47, 0.17) | 0.05  (-0.30, 0.40) | -0.33  (-0.72, -0.01) |
|  | Carbohydrate, iv | 0.38  (-0.30, 1.07) | 0.26  (-0.39, 0.92) | 0.18  (-0.67, 1.01) | 0.46  (-0.25, 1.22) | 0.46  (-0.25, 1.22) | 2.00  (0.96, 3.04) | 1.61  (0.48, 2.69) |
| Postoperative Fins**¶** | Low-dose carbohydrate | -5.48  (-10.83, -0.18) | -4.42  (-9.21, 0.37) | -5.74  (-11.77, 0.19) | -4.91  (-12.74, 2.79) | -4.39  (-9.31, 0.55) | -2.11  (-9.15, 4.83) | -2.11  (-9.15, 4.83) |
|  | High-dose carbohydrate | -2.46  (-6.42, 1.4) | -1.42  (-4.95, 2.10) | -2.78  (-7.54, 1.91) | -1.94  (-8.77, 4.79) | -1.43  (-5.08, 2.19) | 1.81  (-4.13, 7.53) | -0.97  (-5.11, 3.16) |
|  | Carbohydrate, iv | 12.50  (0.90, 24.1) | 13.56  (1.22, 25.97) | 11.66  (-2.73, 26.04) | 12.50  (0.56, 24.45) | 13.02  (-0.87, 26.94) | 12.51  (2.67, 22.27) | 9.72  (-1.73, 21.29) |
| the serum levels of CRP**¶** | Low-dose carbohydrate | 5.83  (-31.11, 45.84) | -14.25  (-50.60, 22.64) | 11.27  (-28.4, 53.31) | -10.69  (-68.5, 49.63) | -18.03  (-56.23, 20.5) | 14.53  (-17.15, 49.41) | -22.24  (-52.41, 8.54) |
|  | High-dose carbohydrate | -1.42  (-28.28, 27.30) | -21.28  (-46.71, 1.84) | 9.06  (-28.58, 47.45) | -12.73  (-55.16, 29.29) | -20.03  (-46.00, 3.65) | 4.45  (-24.36, 35.30) | -32.19  (-56.81,-8.75) |
| Postoperative pain**¶** | Low-dose carbohydrate | -5.48  (-10.83, -0.18) | -1.52  (-6.32, 3.25) | -0.50  (-6.77, 5.75) | -1.37  (-10.69, 8.01) | -1.80  (-8.13, 4.57) | - | - |
|  | High-dose carbohydrate | -0.41  (-5.20, 4.34) | -1.16  (-3.68, 1.29) | 0.20  (-9.2, 9.48) | -0.69  (-7.02, 5.63) | -1.11  (-3.97, 1.69) | - | - |
|  | Carbohydrate, iv | 0.50  (-6.08, 7.13) | -0.25  (-5.84, 5.34) | 0.90  (-8.01, 9.84) | 0.03  (-9.35, 9.52) | -0.40  (-6.79, 5.98) | - | - |
| Postoperative patient satisfaction**¶** | Low-dose carbohydrate | 5.25  (-2.00, 12.50) | 3.26  (-1.95, 8.46) | 5.25  (-2.00, 12.50) | - | 3.26  (-1.95, 8.46) | 5.25  (-2.00, 12.50) | 3.26  (-1.95, 8.46) |
|  | High-dose carbohydrate | 4.00  (-1.02, 9.01) | 2.00  (-2.99, 7.04) | 4.00  (-1.02, 9.01) | - | 2.00  (-2.99, 7.04) | 4.00  (-1.02, 9.01) | 2.00  (-2.99, 7.04) |
| Postoperative thirst**¶** | Low-dose carbohydrate | -0.90  (-9.14, 7.43) | -3.35  (-14.46, 7.68) | -0.90  (-9.14, 7.43) | - | -3.35  (-14.46, 7.68) | - | - |
|  | High-dose carbohydrate | 0.59  (-6.78, 8.04) | -1.87  (-5.61, 1.85) | 0.59  (-6.78, 8.04) | - | -1.87  (-5.61, 1.85) | - | - |
| Postoperative hungry**¶** | Low-dose carbohydrate | -0.69  (-8.46, 7.07) | -2.24  (-12.64, 8.2) | -0.69  (-8.46, 7.07) | - | -2.24  (-12.64, 8.2) | - | - |
|  | High-dose carbohydrate | 0.43  (-6.52, 7.35) | -1.13  (-4.64, 2.34) | 0.43  (-6.52, 7.35) | - | -1.13  (-4.64, 2.34) | - | - |
| Postoperative anxiety**¶** | Low-dose carbohydrate | 0.09  (-8.59, 8.80) | -2.52  (-14.48, 9.46) | 0.09  (-8.59, 8.80) | - | -2.52  (-14.48, 9.46) | - | - |
|  | High-dose carbohydrate | -0.11  (-8.25, 8.02) | -2.72  (-8.88, 3.45) | -0.11  (-8.25, 8.02) | - | -2.72  (-8.88, 3.45) | - | - |
| Postoperative nausea and vomit**¶** | Low-dose carbohydrate | -1.78  (-4.12, 0.53) | -1.36  (-3.43, 0.72) | -1.78  (-4.12, 0.53) | - | -1.36  (-3.43, 0.72) | - | - |
|  | High-dose carbohydrate | -0.76  (-1.76, 0.16) | -0.35  (-1.16,0.46) | -0.76  (-1.76, 0.16) | - | -0.35  (-1.16,0.46) | - | - |
|  | Carbohydrate, iv | -1.52  (-3.92, 0.84) | -1.10  (-3.24, 1.05) | -1.52  (-3.92, 0.84) | - | -1.10  (-3.24, 1.05) | - | - |
| Postoperative fatigue**¶** | Low-dose carbohydrate | -0.70  (-3.65, 2.26) | -1.49  (-6.53, 3.12) | - | - | - | - | - |
|  | High-dose carbohydrate | 0.00  (-3.06, 3.08) | -0.81  (-3.23,1.25) | - | - | - | - | - |
| Postoperative weakness**¶** | Low-dose carbohydrate | - | - | - | - | - | - | - |
|  | High-dose carbohydrate | 0.68  (-0.69, 2.12) | 0.37  (-0.56, 1.47) | 0.68  (-0.69, 2.12) | - | 0.37  (-0.56, 1.47) | 0.68  (-0.69, 2.12) | 0.37  (-0.56, 1.47) |
| Occurrences of postoperative infection**#** | Low-dose carbohydrate | -0.54  (-1.78, 0.66) | **0.42**  **(0.20,0.81)** | 0.56  (0.13,2.70) | 0.61  (0.10,3.10) | **0.41**  **(0.18,0.97)** | 0.58  (0.17,1.90) | 0.41  (0.19,0.90) |
|  | High-dose carbohydrate | 0.93  (0.42,1.70) | 0.71  (0.37,1.30) | 0.85  (0.24,3.00) | 0.94  (0.24,2.50) | 0.63  (0.23,1.60) | 0.95  (0.41,1.80) | 0.67  (0.27,1.50) |

¶: mean difference and 95% confidence intervals(Crls)；

HOMA-IR: homeostasis model assessment-insulin resistance; FPG: fasting plasma glucose; Fins: fasting insulin level; CRP: C-reactive protein.

#: Odds ratios(ORs)；-: No data available for this outcome.

2. Supplementary Figures

**2.1 Supplementary Figure S1.** Risk of Bias , showing the domain assessment for individual trials


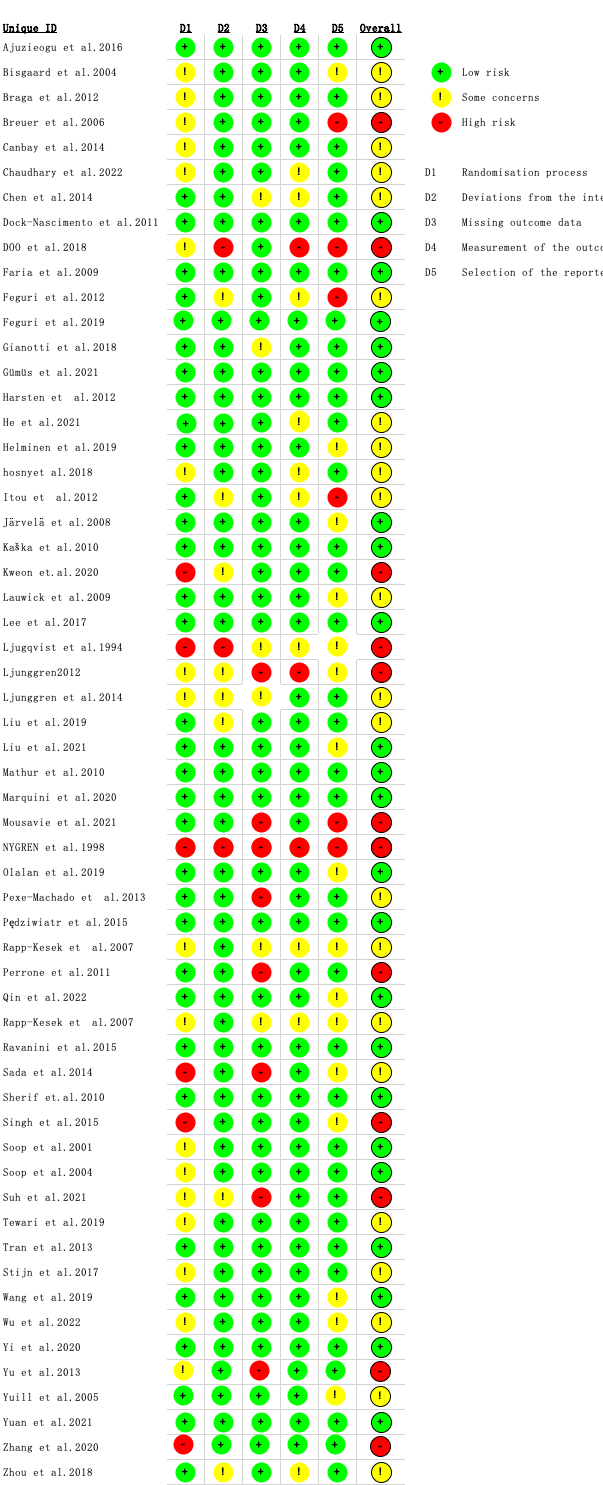


**2.2 Supplementary Figure S2.** Network Plot for The Estimates of Different Interventions on IR of Postoperative Patients That Excluded Trials at High Risk of Bias and data for the imputation methods.

The area of every circle is proportional to the number of randomly assigned patients and indicates the sample size. The width of the lines is proportional to the number of trials that directly compared the two medications.

**2.3 Supplementary Figure S3.** Comparison-adjusted Funnel plot


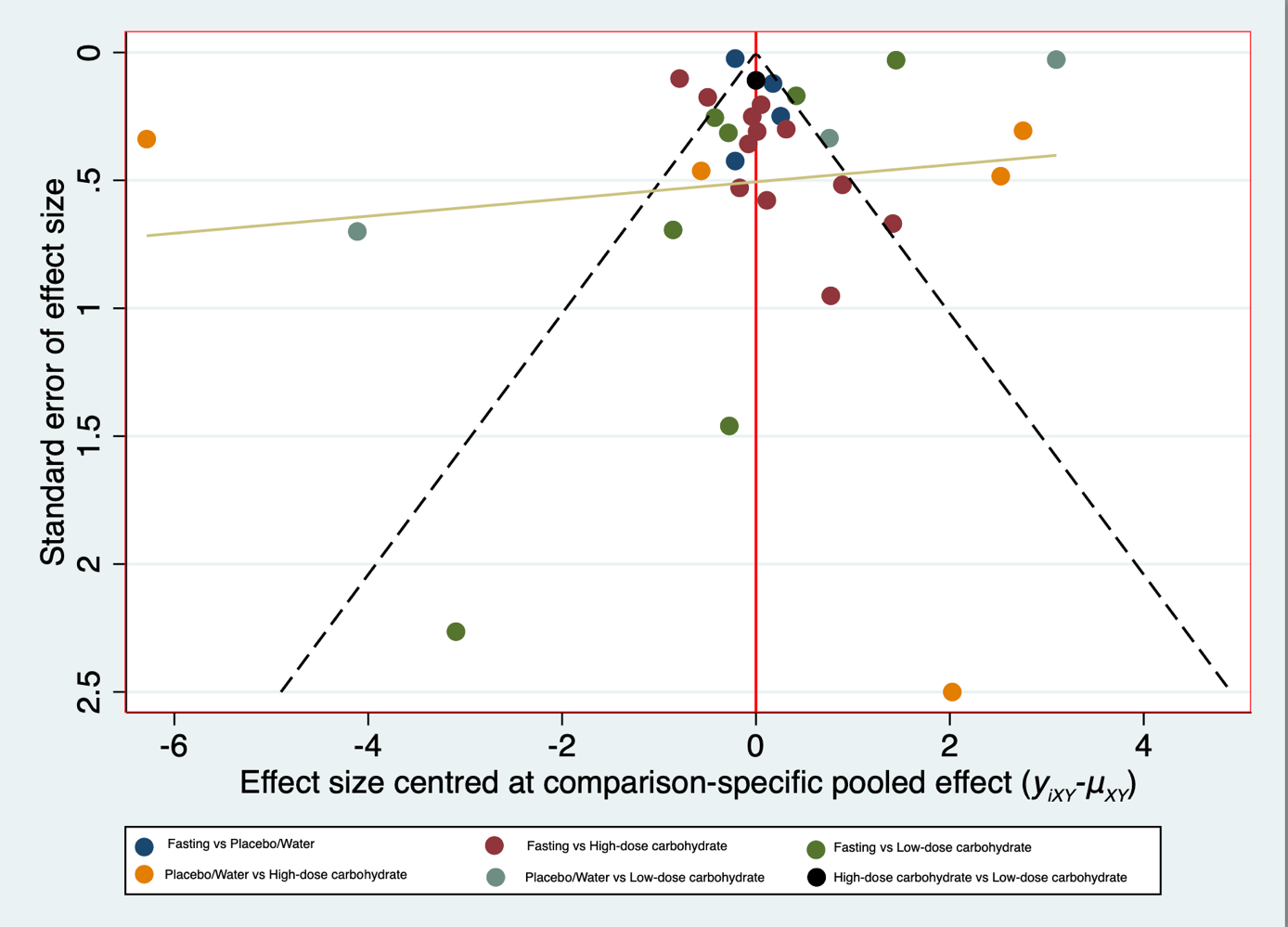


Null hypothesis is represented by the zero central line, which indicates there is no difference between comparison-specific pooled estimates and study-specific estimates. different colored points indicate different comparisons.

**2.4 Supplementary Figure S4.** Network plot for different second outcomes

The area of every circle is proportional to the number of randomly assigned patients and indicates the sample size. The width of the lines is proportional to the number of trials that directly compared the two medications.

(A): residual gastric volume (RGV) during the operation; (B): insulin sensitivity; (C): postoperative fasting plasma glucose; (D): postoperative fasting serum insulin; (E): the serum levels of C-reactive protein (CRP) within the first 24h after surgery; (F): postoperative pain; (G): postoperative patients’ satisfaction; (H): postoperative thirst; (I): postoperative hunger; (J): postoperative anxiety; (K): postoperative nausea and vomit (PONV); (L): postoperative weakness;(M): the occurrence of postoperative infection.
